# MSC-EVs attenuate subretinal fibrosis in choroidal neovascularization through miR-21-5p-mediated inhibition of EMT and MMT and suppression of inflammation

**DOI:** 10.1186/s12974-026-03836-w

**Published:** 2026-04-30

**Authors:** Xiang-Ling Yuan, David Hughes, Xuexue Cui, Ruiqi Zhou, Jinyan Qi, Caijiao Yi, Jian Liu, Sarah McFetridge, Johnatas Dutra Silva, Hojjat Naderi-Meshkin, Anna D Krasnodembskaya, Heping Xu, Mei Chen

**Affiliations:** 1 Aier Academy of Ophthalmology, Central South University, Changsha, Hunan 410004 China; 2 Aier Eye Institute, Changsha Aier Eye Hospital, Changsha, Hunan 410004 China; 3 The Wellcome-Wolfson Institute for Experimental Medicine, Queen’s University Belfast, Belfast, UK

**Keywords:** Mesenchymal stem cell, Extracellular vesicles, Subretinal fibrosis, Epithelial-mesenchymal transition, Macrophage-to-myofibroblast transition, Immunomodulation, Age-related macular degeneration

## Abstract

**Background:**

Subretinal fibrosis causes irreversible vision loss in neovascular age-related macular degeneration (nAMD). Sustained macular inflammation drives the initiation and progression of fibrosis by activating profibrotic cells and perpetuating tissue damage. This study investigated the therapeutic potential of mesenchymal stem cell-derived extracellular vesicles (MSC-EVs) in mitigating nAMD-associated subretinal fibrosis.

**Methods:**

MSC-EVs were prepared from human bone marrow-derived MSCs and characterized using nanoparticle tracking analysis, transmission electron microscopy, and Western Blotting. Subretinal fibrosis was induced in C57BL/6J mice using the two-stage laser-induced model. MSC-EVs were injected either intravitreally (1 × 10^8^ particles/eye, single injection) or retro-orbitally (1 × 10^8^ particles, two injections four days apart) immediately after the second laser. Eyes were collected 10 days post-second laser for immunostaining of collagen-1 and CD31 or iso-lectin B4. In vitro, primary human RPE and ARPE-19 cells were treated with TGF-β2 (10 ng/mL) to induce epithelial-mesenchymal transition (EMT); peritoneal macrophages were treated with TGF-β1 (10 ng/mL) to induce macrophage-to-myofibroblast transition (MMT). After 48 h, cells were treated with MSC-EVs (cell-to-MSC-EV ratio = 1:2000) for 3 days. Myofibroblast markers (αSMA, fibronectin, and collagen-1) were examined by immunocytochemistry and quantitative PCR (qPCR). Human iPCS-derived macrophages (iMACs), bone-marrow-derived macrophages, peritoneal macrophages, and BV2 microglia were treated with LPS (100 ng/mL) and IFN-γ (20 ng/mL) for 24 h with or without MSC-EVs (1:2000). Small RNA sequencing was used to identify specific functional molecules within MSC-EVs. Immune-related gene expressions were evaluated by qPCR.

**Results:**

Intravitreal and retroorbital administration of MSC-EVs reduced collagen-1^+^ fibrotic lesions by 46% and 30%, respectively, and significantly inhibited infiltrating Iba-1^+^ cells. In vitro, MSC-EVs attenuated TGF-β2-induced upregulation of αSMA, fibronectin, and collagen-1 at both protein and mRNA levels in RPE cells. Similarly, the expression of *Acta2*, *Fn1*, and *Col1a1* in TGF-β1-treated macrophages was also significantly reduced following MSC-EV treatment. In LPS + IFN-γ-stimulated immune cells, MSC-EVs significantly suppressed the expression of *Il6* and *Il1b* in all cell types, and reduced the expression of *Inos*, *Tnfa*, and *Cd86* in iMACs, peritoneal macrophages, and BV2 cells. Enriched hsa-miR-21-5p was identified in MSC-EVs and involved in the TGF-β-related signaling pathway. Overexpression of miR-21-5p mimic abrogated the TGF-β1-driven upregulation of pro-fibrotic markers in RPE and macrophages.

**Conclusions:**

Local administration of MSC-EVs effectively mitigated subretinal fibrosis and reduced inflammation in the mouse model of nAMD, potentially via miR-21-5p-mediated attenuation of EMT and MMT, and suppression of inflammation. MSC-EVs represent a novel cell-free therapeutic strategy for macular fibrosis in nAMD.

**Supplementary Information:**

The online version contains supplementary material available at 10.1186/s12974-026-03836-w.

## Background

Fibrosis, characterized by excessive extracellular matrix (ECM) deposition and tissue remodeling, is a hallmark of numerous chronic diseases, contributing to progressive organ dysfunction [1, 2]. In ocular pathology, fibrosis underlies vision loss in age-related macular degeneration (AMD), a leading cause of irreversible blindness in individuals over 50 years of age [3, 4]. Neovascular AMD (nAMD) is characterized by the ingrowth of abnormal blood vessels into the sub-retinal pigment epithelial (RPE) and subretinal spaces of the macula, culminating in subretinal fibrotic plaque formation [4–6]. Central to this process is the epithelial-mesenchymal transition (EMT) of RPE cells, a cellular reprogramming event whereby epithelial cells acquire mesenchymal phenotypes, marked by reduced Zonula Occludens-1 (ZO-1) expression and upregulation of vimentin and alpha-smooth muscle actin (α-SMA) [4, 7, 8]. EMT has previously been shown to occur in RPE cells when exposed to factors such as transforming growth factor-beta (TGF-β) [8, 9], tumor necrosis factor-alpha (TNF-α) [10], or complement proteins such as C5a [11]. Additionally, inflammation plays a pivotal role in nAMD-associated fibrosis, through both complement system interactions along with immune cell infiltration, including T/B cells and macrophages [11–13]. Recent evidence suggests that infiltrating macrophages can undergo macrophage-mesenchymal transition (MMT), a process analogous to EMT that enhances their fibrogenic potential through ECM production and pro-fibrotic cytokine release [12, 14]. The interplay of EMT, MMT, and inflammation underscores the complexity of subretinal fibrosis, necessitating novel therapeutic strategies to mitigate these processes.

Mesenchymal stem cells (MSCs), derived from sources such as bone marrow, adipose tissue, or umbilical cord, are recognized for their immunomodulatory, anti-inflammatory, and regenerative properties [15–18]. However, clinical applications of MSCs are limited by challenges, including immune rejection and variable engraftment [19, 20]. Extracellular vesicles (EVs) released by MSCs (MSC-EVs) have emerged as a promising cell-free alternative, delivering bioactive cargos including proteins, lipids, and microRNAs (miRNAs) to modulate recipient cell behavior [21]. MSC-EVs exhibit potent anti-fibrotic activities in multiple organs, including the liver, skin, heart, and lung, by attenuating ECM deposition, suppressing inflammation, and promoting tissue repair [22–25]. These findings suggest that MSC-EVs may hold therapeutic promise for ocular fibrosis, particularly in nAMD-mediated subretinal fibrosis. The efficacy of MSC-EV is underpinned by their capacity to modulate key pro-fibrotic signaling pathways. Furthermore, MSC-EVs exert robust immunomodulatory effects by polarizing macrophages toward an anti-inflammatory M2 phenotype, decreasing pro-fibrotic cytokines such as interleukin-1 beta (IL-1β), and TNF-α, and mitigating inflammation [26–29]. The concurrent targeting of both inflammation and myofibroblast activation positions MSC-EVs as a potential therapeutic modality for subretinal fibrosis.

Here, we investigate the hypothesis that MSC-EVs can mitigate subretinal fibrosis by attenuating TGF-β-induced EMT and MMT. Additionally, we assess their therapeutic effects in the murine model of two-stage laser-induced subretinal fibrosis [30]. By concurrently targeting the core pathways of EMT, MMT, and associated inflammation, MSC-EVs offer a novel cell-free strategy to mitigate subretinal fibrosis in AMD.

## Materials and methods

### Culture of mesenchymal stem cells

Human bone marrow-derived mesenchymal stem cells (MSCs) were obtained from RoosterBio (Cat. MSC-031, RoosterBio, Frederick, MD) at Passage 0 (P0). The cells were cultured in T75 flasks using RoosterNourish-MSC-XF medium (Cat. KT-016, RoosterBio) for the initial passage (P0 – P1). The cells were maintained under standard conditions (21% O2, 5% CO2) and passaged upon reaching 80–90% confluence to prevent cellular quiescence. From Passage 1 (P1) onward, MSCs were cultured in serum-free medium, and their supernatants (conditioned media) were collected every 2–3 days. Passages 2 ~ 4 of MSCs were used in the study.

### Extracellular vesicle (EV) isolation and characterization

**Fig. 1:**
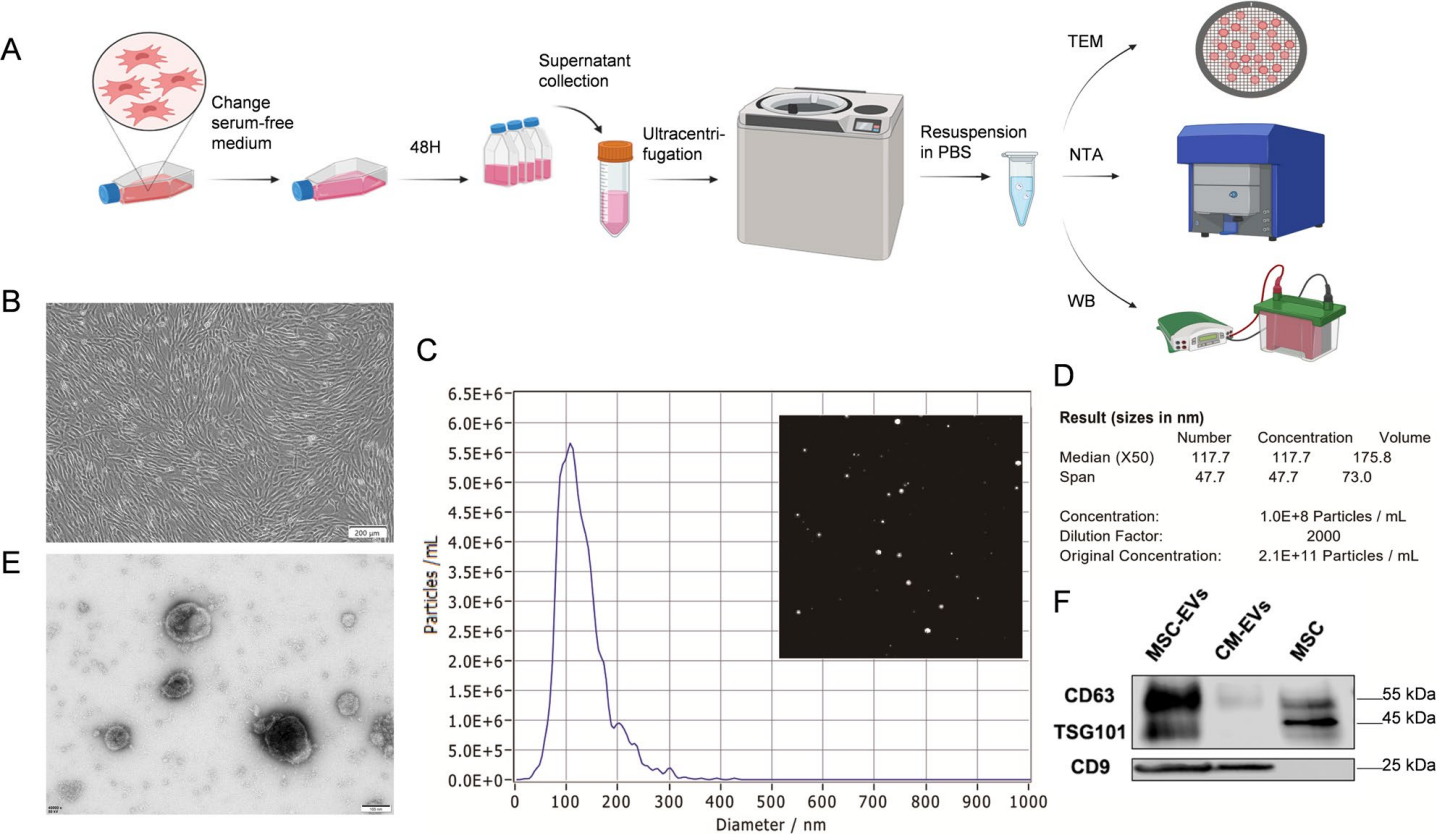
Extraction andcharacterization of extracellular vesicles (EVs) from human bone marrow-derived mesenchymal stem cells (hBM-MSCs). **A** Schematic diagram ofcell culture and extracellular vesicle extraction. **B** Primary hBM-MSCs exhibited a typical fibroblast-like, spindle-shaped morphology in opticalmicroscope. Scale bar =200 μm. **C** The size and concentration of EV particles were assessed by nanoparticle tracking analysis (NTA), and theirmovement under Brownian motion was captured. **D** The total number of particles from NTA in C was calculated. The median and span valueshown the particle size in the 50-200 nm range and the original concentration of particles was computed. **E** Transmission electron microscopy(TEM) was utilized to analyze morphology of EVs. Isolated EVs exhibited typical cup-shaped or saucer-like morphology with a bilayer membranestructure. Scale bar = 100 nm. **F** Western blot analysis detected the presence of EV-associated protein markers CD9, CD63, and TSG101

EVs were isolated using differential ultracentrifugation as previously described [31, 32]. Briefly, the conditioned medium was sequentially centrifuged at 300 × *g* for 10 min at 4 °C, followed by 2000 × *g* for 30 min and 10,000 × *g* for 30 min at 4 °C to eliminate cells, cellular debris and apoptotic bodies, respectively. The resulting supernatants were ultracentrifuged at 110,000 × *g* for 120 min at 4 °C using an Optima XE-100 ultracentrifuge equipped with an SW32Ti rotor (Beckman Coulter, Pasadena, CA). The EV-containing pellets were resuspended in phosphate-buffered saline (PBS), washed, and subjected to a second ultracentrifugation under the same conditions. Final EV pellets were resuspended in PBS (Fig. 1A).

We measured the EVs’ particle size and concentration using nanoparticle tracking analysis (NTA) with Zetaview-PMX120-Z (Particle Metrix, Inning am Ammersee, Germany) following the manufacturer’s instructions. In brief, the EVs were diluted in 1X PBS buffer. The particles were illuminated by a 520 nm laser, and their Brownian motion was captured, recorded, and analyzed at 11 positions. The ZetaView system was calibrated using 100 nm polystyrene particles. Each sample was measured in triplicate.

The morphology of EV was examined by Transmission Electron Microscopy (TEM) (JEM1400; JEOL Co. Ltd., Tokyo, Japan). The samples were fixed in glutaraldehyde, post-fixed in osmium tetroxide, dehydrated in a graded ethanol series, and embedded in epoxy resin. Ultra-thin sections (70 nm) were stained with uranyl acetate and lead citrate. Imaging was performed at an acceleration voltage of 80 kV and captured at a magnification of 10000x. EV protein markers, including CD9, CD63, and TSG101, were confirmed by Western blotting.

### Animals and induction of subretinal fibrosis

C57BL/6J mice (20–22 g; 6–8 weeks old) were purchased from Hunan SJA Laboratory Animal Co., Ltd (Changsha, China) or the Charles River Laboratories and maintained in a specific pathogen-free (SPF) condition with a 12-hour light/dark cycle, free access to food and water in a temperature-controlled room. All animal-related procedures were conducted following the Association for Research in Vision and Ophthalmology (ARVO) Statement for the Use of Animals in Ophthalmic and Vision Research. The protocols were approved by the Animal Welfare Ethics Committee of AIER Eye Institute (Ref no: AEI20230016) and the Animal Welfare and Ethical Review Body (AWERB) of Queen’s University Belfast (License number 2876, approved 28/10/2019).

A two-stage laser protocol, detailed previously [30, 33], was used to induce subretinal fibrosis in mice. Briefly, mice were anaesthetized with an intraperitoneal injection of a mixture of Zoletil (75 mg/kg, Virbac, Carros, France) and xylazine (10 mg/kg, Sigma-Aldrich, St. Louis, MO). Pupils were dilated with 0.5% tropicamide and 0.5% phenylephrine (Santen Pharmaceutical Co., Ltd., Osaka, Japan). Carboxymethylcellulose Sodium Eye Drops (Allergan Pharmaceutical Co., Ltd., Dublin, Ireland) were used to moisten the ocular surface. The eyes received four laser burns at the two optic disc diameters away from the optic nerve head using a Photocoagulator (EASYRET; Quantel MEDI, France; wavelength = 577 nm, power = 100 mW, exposure time = 150 ms, single spot diameter = 100 μm). Seven days later, a second laser burn with the same laser parameters was conducted on each pre-existing CNV lesion. Eyeballs were collected 10 days after the second laser and processed for further analysis.

### Intravitreal and retroorbital injection of extracellular vesicles

Mice were anaesthetized and their pupils were dilated before injection. Two microliters of MSC-EVs (1 × 10^8^ particles) or vehicle (PBS) was slowly injected into the vitreous cavity using a Hamilton syringe immediately after the second laser under a stereomicroscope (OLYMPUS SZ61, Olympus Corporation, Tokyo, Japan). For retroorbital injection, fifty microliters of EV suspension (1 × 10^8^ particles) or PBS were injected into the retroorbital sinus using a 33-gauge needle immediately after the second laser, followed by a second injection four days later. Six days after the second injection, fundus photography and fluorescence angiography were performed. Mouse eyes were enucleated and fixed in 2% paraformaldehyde (PFA; Cat. 158127, Sigma-Aldrich) for 2 h and processed for RPE/choroid flatmount or cryosection investigations.

### RPE cell culture and EMT induction

ARPE-19 cells (Cat. CRL-2302, ATCC, Manassas, VA) were maintained in DMEM/F12 medium (Cat. 11330032, Thermo Fisher Scientific, Waltham, MA), supplemented with 10% fetal bovine serum (FBS; Cat. 10270106) under standard conditions. Primary human RPE cells (phRPE; Cat. 6540, ScienCell, Carlsbad, CA) were cultured on plates coated with Poly-L-lysine (Cat. 4707-50 ml, Sigma-Aldrich, Gillingham, UK) using epithelial cell medium (EpiCM; Cat. 4101) supplemented with 2% FBS (Cat. 0010), epithelial cell growth supplement (Cat. 4152), and penicillin/streptomycin solution (Cat. 0503), all from ScienCell (ScienCell Research Laboratory, Carlsbad, CA). Cells were maintained under standard conditions and used between passages 1 and 3 (P1–P3).

For experiments, 2 × 10^4^ ARPE-19 or phRPE cells were seeded onto designated experimental plates as described in the respective experimental protocols. After 24 h, the culture medium was switched to DMEM/F12 containing 1% FBS for ARPE-19 or EpiCM with 1% FBS for phRPE cells for the duration of the experiment. To induce EMT, cells were treated with 10 ng/mL TGF-β2 (Cat. 7346-B2-005/CF, R&D Systems, Minneapolis, MN) or vehicle control (4 mM HCl with 0.1% bovine serum albumin, BSA) for 48 h. Subsequently, cells were exposed to EVs at a cell-to-EV particle ratio of 1:2000 for an additional 3 days and subjected to morphological and EMT-related marker detections. PBS served as the vehicle control for EV treatments.

### Generation and treatment of iPSC-derived macrophages (iMacs)

Human iPSCs (~ 80% confluency) were dissociated into small clumps (3–10 cells) and cultured in ultra-low attachment 6-well plates (Cat. CLS3471, CORNING COSTAR, Corning, NY) using aggregation medium composed of KnockOut DMEM/F12 (Cat. 12660012), 10% KnockOut Serum Replacement (Cat. 10828028), 1× non-essential amino acids (Cat. 11140050), GlutaMAX (Cat. 35050061), penicillin/streptomycin (Cat. 15140122), β-mercaptoethanol (55 µM) (Cat. 21985023), (all from Thermo Fisher Scientific), and ROCK inhibitor Y-27,632 (50 µM) (Cat. HY-10071, CliniSciences, Nanterre, France) to form embryoid bodies (EBs) (Day 0). On Day 1, EBs were transferred into myeloid differentiation medium (50% DMEM/F12 (Cat. 11330032) and 50% E8 medium) supplemented with BMP-4 50 ng/mL (Cat. 120-05), VEGF 50 ng/mL (Cat. 100 − 20), SCF 20 ng/mL (Cat. 300-07) (all from ThermoFisher Scientific), β-mercaptoethanol (55 µM), and penicillin/streptomycin. Six days later, EBs were cultured in priming medium consisting of X-VIVO 15 (Cat. 02-053Q, Lonza, Basel, Switzerland), β-mercaptoethanol, VEGF 50 ng/mL, SCF 20 ng/mL, M-CSF 100 ng/mL (Cat. 300 − 25, ThermoFisher Scientific), and IL-3 25 ng/mL (Cat. 200-03, Thermo Fisher Scientific) for 48 h. Cystic aggregates were transferred to T75 flasks in macrophage-production medium (X-VIVO 15 supplemented with M-CSF 100 ng/mL and IL-3 25 ng/mL), and the cells were harvested for experiments (see below).

iMacs were expanded in macrophage maintenance medium (X-VIVO 15 with M-CSF 50 ng/mL). Phenotypic validation was performed by flow cytometry using established macrophage surface markers. The phenotype and phagocytic activity of iMacs remained stable for at least 14 days post-collection.

For inflammation assessment, iMacs were seeded for 24 h and administered with fresh macrophage maintenance medium containing 20 ng/mL recombinant human interferon-γ (IFN-γ) (Cat. PHC403, Gibco, Waltham, MA) and 10 ng/mL lipopolysaccharides (LPS) (Cat. L2880-10MG, Sigma-Aldrich) along with EVs for an additional 24 h.

### Generation and treatment of peritoneal macrophages (PMs)

Peritoneal macrophages were isolated and cultured as previously described [34, 35]. In brief, C57BL/6J mice were intraperitoneally injected with 2 mL of 3% (w/v) thioglycolate medium (T9032, Sigma-Aldrich). After 3 days, mice were euthanized, and macrophages were harvested by lavaging the peritoneal cavity with cold DMEM. The cells were collected after centrifugation and cultured in DMEM supplemented with 10% FBS and incubated at 37 °C in a 5% CO₂ incubator for 6 h. The non-adherent cells were removed by washing with PBS. Macrophage phenotypes were validated by flow cytometry.

To induce MMT, PMs were treated with 10 ng/mL of TGF-β1 (Cat. 7666-MB-005/CF, R&D Systems) with or without EVs at a cell-to-EV particle ratio of 1:2000 for 48 h. The cells were then subjected to MMT assessments (see below).

### BV2 and RAW264.7 cell culture and treatment

BV2 and RAW264.7 cells were cultured in high-glucose DMEM (Gibco, USA) supplemented with 10% FBS (Cat. BS-1105, Opcel, Hohhot, China), and 100 IU/mL penicillin and 100 µg/mL streptomycin (Cat. 15140122, ThermoFisher Scientific). Upon reaching approximately 90% confluence, cells were detached with 0.25% trypsin-EDTA (Cat. 25200056, ThermoFisher Scientific) and replated for experimentation.

For M1 polarization, BV2, RAW264.7 cells, and PMs were seeded at equal densities in 12-well plates. When reaching 80–90% confluence, the cells were stimulated with LPS (100 ng/mL) and IFN-γ (20 ng/mL) for 24 h with or without EVs. Twenty-four hours later, cells were harvested for qRT-PCR analysis.

### Morphological assessment

RPE cells were imaged using a Nikon 6D live cell imager (Nikon, Michigan, USA) at two time points: Day 0 (D0), 48 h after TGF-β2 induction (i.e., Day 3, D3), post EV induction. Fifty cells per sample were randomly selected using a grid-based numbering system superimposed on the images. Cell area and perimeter was measured and calculated using ImageJ. Cell circularity was determined using the formula: circularity = (4 × π × Area) / Perimeter².

### Small RNA sequencing library construction and bioinformatics analysis

Total RNA was extracted from the purified MSC-EVs by Trizol reagent kit (Invitrogen, California, USA), and the RNA in a size range of 18-30nt were enriched by polyacrylamide gel electrophoresis (PAGE). Then the 3’ and 5’ adapters were successively ligated to the RNAs. The ligation products were reverse transcribed by polymerase chain reaction (PCR) amplification and the 140–160 bp size PCR products were enriched to generate a cDNA library and sequenced using Illumina NovaSeq X Plus by Gene Denovo Biotechnology Co. (Guangzhou, China).

For the bioinformatics analysis, raw sequencing reads underwent stringent quality control, and adapter sequences along with low-quality bases were trimmed utilizing Cutadapt (version 4.9; Martin, 2011). The resulting high-quality clean reads were mapped to the reference genome and the miRBase database utilizing the bowtie software (version 1.1.2; Langmead et al., 2009). The miRNA expression level was calculated and normalized to transcripts per million (TPM).

### Target gene prediction and functional enrichment analysis

Based on the sequences of identified miRNA, the candidate target genes were predicted by strictly intersecting the output of three independent databases: RNAhybrid, miRanda, Targetscan. Subsequently, the consensus target genes were subjected to Gene Ontology (GO) functional annotation and Kyoto Encyclopedia of Genes and Genomes (KEGG) pathway enrichment analysis. A hypergeometric test was applied, and the calculated p-value was gone through False Discovery Rate (FDR) Correction, taking FDR ≤ 0.05 as a threshold. The topological networks and functional enrichments were visualized using Sankey diagrams, hierarchical clustering heatmaps, and enrichment bubble plots via the analysis platform (https://www.omicshare.com/).

### Transfection of miRNA mimics

Cells were cultured on a 12-well plate at an appropriate density one day in advance. The miRNA mimics (5’-3’: UAGCUUAUCAGACUGAUGUUGA; 3’-5’: AACAUCAGUCUGAUAAGCUAUU; Sangon Biotech, China) or the same concentration of negative control (mimic NC) (5’-3’: UUGUACUACACAAAAGUACUG; 3’-5’: GUACUUUUGUGUAGUACAAUU; Sangon Biotech, China) was subsequently transfected into these cells by Lipofectamine^®^ 2000 Reagent (Cat. 11668-019, Invitrogen). The medium was changed 8 h later. In parallel, TGF-β1 or LPS + IFN-γ was added to stimulate cells for EMT/MMT or M1. After treatment, the miRNA mimic transfection efficiency and fibrotic-related or pro-inflammatory genes expression in cells were detected.

### RNA extraction and quantitative real‑time PCR (qRT‑PCR)

Total RNA was isolated using SteadyPure Quick RNA Extraction Kit (Cat. AG21023, Accurate Biology, Changsha, China) or the Maxwell RSC simplyRNA Cells Kit (Cat. AS1390, Promega, Seattle, WA) following the manufacturers’ instructions. cDNA was synthesized using HiScript II Q RT SuperMix (Cat. R223, Vazyme, Nanjing, China) or the High-Capacity RNA-to-cDNA Kit (Cat. 4387406, Thermo Fisher Scientific). The expression level of genes of interest was determined by qRT‑PCR using ChamQ Universal SYBR qPCR Master Mix (Cat. Q711, Vazyme) on LightCycler^®^480 Instrument II (Roche, Germany). The miRNAs were acquired by SteadyPure Tissue and Cell Small RNA Extraction Kit (Cat. AG21027, Accurate Biology) and miRNA 1st strand cDNA synthesis kit (Cat. AG11717, Accurate Biology). A synthetic exogenous Caenorhabditis elegans miRNA (cel-miR-39) was added to the samples as a spike-in control during RNA extraction to normalize for reverse transcription efficiency. mRNA expression levels of target genes were calculated using the delta delta Ct method and normalized to the housekeeping gene *Gapdh*, * 18S*,* RPL11*, and *GAPDH* respectively. The primers of genes were synthesized by TsingkeBiotechnology (Changsha, China), and the sequences can be found in Supplementary Table 1.

### Immunofluorescent staining

RPE/choroid flatmounts, cryosections and fixed cells by PFA were blocked and permeabilized with 10% goat serum and 0.1% Triton X-100 in BSA at room temperature (RT) for 2 h, followed by incubating with primary antibodies (Supplementary Table 2) overnight at 4 °C. After thorough washes, the samples were incubated with fluorophore-conjugated secondary antibodies (Supplementary Table 3) and DAPI at RT for 2 h in the dark. All samples were mounted with an anti-fade medium and imaged using a confocal microscope (Zeiss, Braunschweig, Germany). The quantity of lesion area was performed through ImageJ automated threshold-based segmentation.

#### EV staining

EVs were fluorescently labelled using the ExoGlow™-Protein EV Labelling Kit (Cat. EXOGP300A-1, System Biosciences, Palo Alto, CA). The EV pellet was reconstituted in PBS and 1 μL of 500X fluorescent labeling dye was added to 500 μL of EV suspension. The mixture was incubated at 37°C with continuous agitation at 350 rpm for 20 min using an orbital shaker. 167 μL of ExoQuick-TC reagent was added to the suspension and incubated at 4°C overnight. The EV suspension was centrifuged at 10,000 rpm for 10 min, and the pellet was resuspended in PBS for subsequent experiments.

### Flow cytometry analysis

For iMACs experiments cells were harvested from 6-well plates on ice. The cells were centrifuged at 400 × *g* for 5 min at 4 °C, resuspended in 100 µL Invitrogen™ eBioscience™ Flow Cytometry Staining Buffer (Cat. 15346785, ThermoFisher Scientific), and incubated with anti-human Fc Receptor antibody (Supplementary Table 2). The samples were then incubated with fluorochrome-conjugated single stain flow antibodies (Supplementary Table 2) for 30 min at 4 °C in the dark. After thorough washes, samples were analysed using a BD FACSCanto II Flow Cytometer (BD Biosciences, Franklin Lakes, NJ). In EV uptake and internalization flow experiments (Supplementary Fig. 2) once staining protocols and administration to recipient donor cells was completed as mentioned previously, donor cells were collected within 1–2 H by cell scraping on ice and fluorescence analyzed using a BD FACSCanto II Flow Cytometer (BD Biosciences, Franklin Lakes, NJ).

### Western blotting

Total protein from MSCs and EVs was extracted using RIPA lysis buffer (Cat. P0013B, Beyotime, Shanghai, China) supplemented with protease and phosphatase inhibitors. Protein concentration was measured using the Micro BCA Protein Assay Kit (Cat. 23235, ThermoFisher Scientific). After denaturation, equal amounts of protein (15 µg) were separated on a 10% SDS-polyacrylamide gel and transferred onto nitrocellulose membranes. The membrane was blocked with 5% skimmed milk in TBST for 1 h at RT, and incubated overnight at 4 °C with the following primary antibodies: anti-CD9, anti-CD63, and anti-TSG101 (Supplementary Table 2), followed by horseradish peroxidase (HRP)-conjugated secondary antibody incubation for 1 h at RT. Immunoreactive bands were visualized using an enhanced chemiluminescence (ECL) detection kit (WBULS0100, Millipore, Boston, MA), and detected using the ChemiDoc™ MP Imaging System (Bio-Rad, Hercules, CA).

### Statistical analysis

Statistical analysis and plotting were performed using GraphPad Prism (version 10; GraphPad Software, San Diego, CA). All data are presented as mean ± standard deviation (SD). Comparisons between two groups were conducted using an independent Student’s t-test. For multiple group comparisons, one-way analysis of variance (ANOVA) followed by Tukey’s post-hoc test for multiple comparison corrections was applied. A *p*-value < 0.05 was considered statistically significant.

## Results

### Characterization of mesenchymal stem cell-derived EVs (MSC-EVs)

Human bone marrow-derived MSCs display spindle-shaped, fibroblast-like morphology under 40× magnification (Fig. 1B). MSC-EVs demonstrated a typical size distribution ranging from 70 to 170 nm in diameter, and the most abundant particle size was 117.7 ± 47.7 nm on NTA investigation (Fig. 1C, D). MSC-EV preparations exhibited a particle concentration of 2.1 × 10¹¹ particles/mL and a total protein concentration of 0.392 µg/µL, corresponding to a protein-to-particle ratio of approximately 1.9 µg per 10⁹ particles. TEM revealed the cup-shaped or saucer-like morphology and a clear bilayer membrane structure (Fig. 1E). Western blot showed the enrichment of canonical EV markers CD9, CD63, and TSG101 in MSC-EVs compared with MSCs and culture medium-derived EVs (CM-EVs) (Fig. 1F).

### MSC-EVs alleviated subretinal fibrosis in two-stage laser-induced mice

**Fig. 2:**
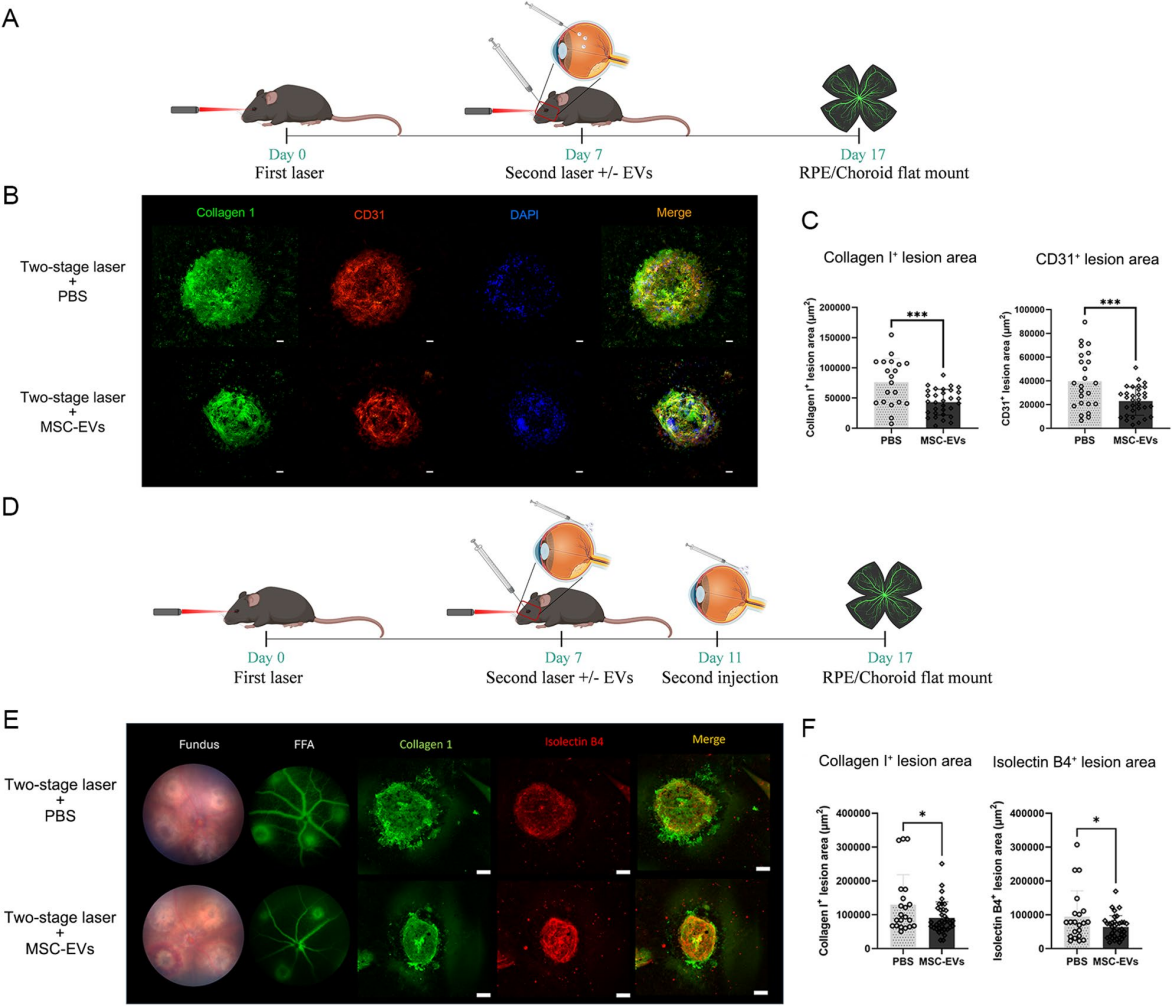
MSC-EVs decreased the lesion area in two-stage laser-induced subretinal fibrosis mice. **A** Schematic view of the study design. Subretinal fibrosis was induced using the two-stage laser-induced protocol. MSC-EVs were injected (intravitreal injection) on the day of the second laser, eyes were collected 10 days after EV treatment for immunofluorescence. **B **Representative immunofluorescence images of RPE-Choroid flat showing collagen-1 (green) and CD31 (red) stained lesion from each group. Scale bar = 20 μm. **C** Quantitative analysis of collagen-1^+^ (fibrotic component) and CD31^+^ lesion area in each group. **D** Administration of retroorbital injection of MSC-EVs in two-stage laser-induced subretinal fibrosis mice. **E** Representative fundus, FFA image, and immunofluorescence images of RPE-Choroid flat showing collagen-1 (green) and isolectin B4 (red) stained lesion from each group. Scale bar = 50 μm. **F** Quantitative analysis of collagen-1^+^ and isolectin B4^+^ lesion area in each group. MSC-EVs: mesenchymal stem cell-derived extracellular vesicles; Data shown as mean ± SD. n = 5 – 6 eyeballs; Independent Student’s t-test, *p<0.05, **p<0.01, ***p<0.001

To evaluate the therapeutic potential of MSC-EVs in subretinal fibrosis, the particles were administered either intravitreally (immediately after the second laser) or intraorbitally on the day of the second laser and four days later (Fig. 2A, D). Lesion size was assessed 10 days after the second laser in RPE/choroid flatmounts (Fig. 2A, D). The results show that intravitreal administration of MSC-EVs reduced the area of collagen-1^+^ subretinal fibrotic lesion by approximately 46% compared with the PBS treatment group (Fig. 2B, C). The treatment also significantly reduced the size of CD31^+^ vascular lesions (Fig. 2D). Similarly, retroorbital injection of MSC-EVs also significantly reduced collagen-1^+^ and Isolectin B4^+^ lesions, although less pronounced (i.e., 30% and 32% reduction in fibrosis and vascular lesions, respectively, Fig. 2E, F) compared to those via intravitreal injection (Fig. 2C, D). Our results suggest that MSC-EVs can successfully alleviate CNV-mediated subretinal fibrosis.

### MSC-EVs inhibited the epithelial-mesenchymal transition from RPE cells

**Fig. 3:**
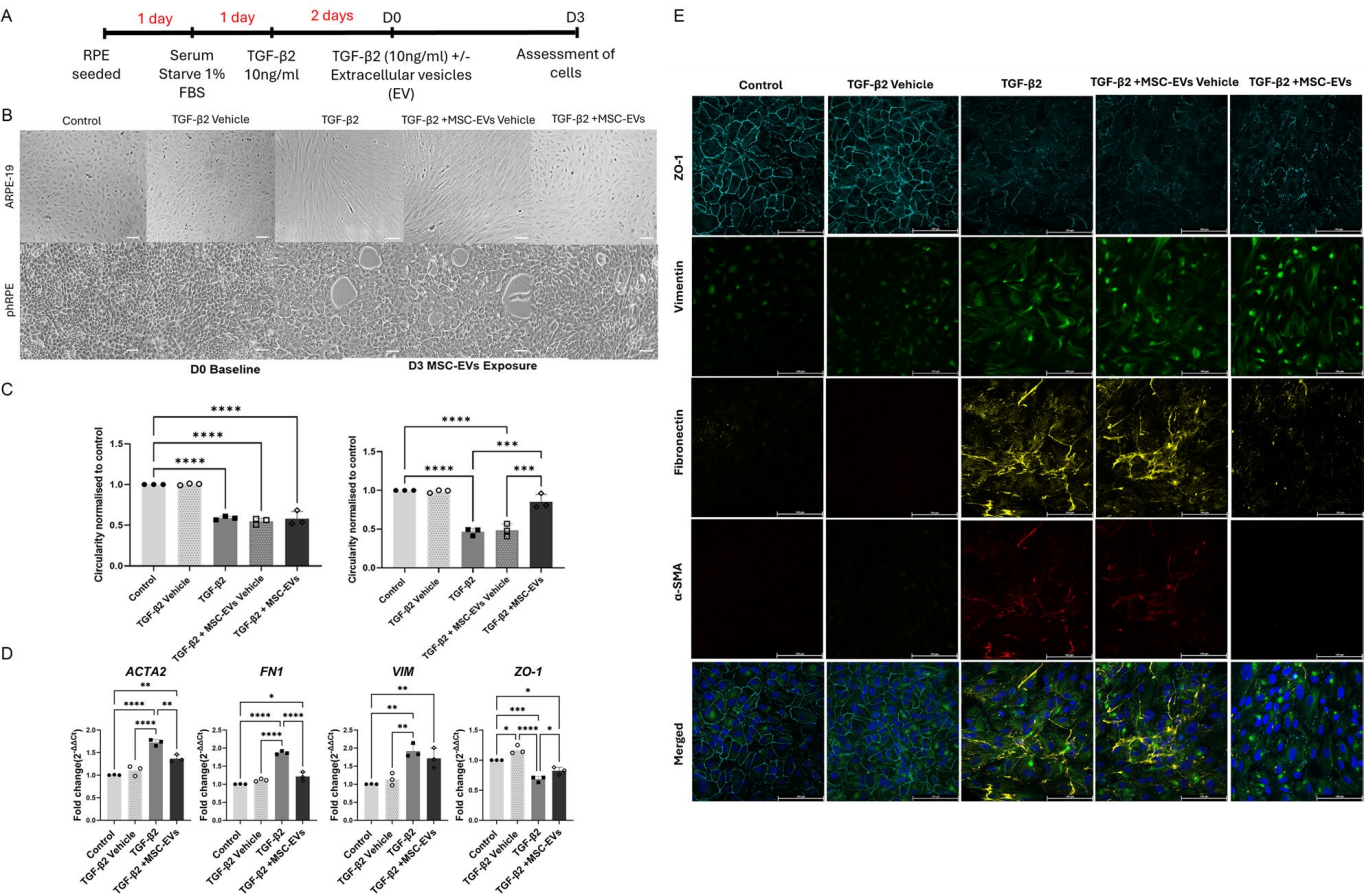
MSC-EVs attenuated TGF-β2 induced epithelial-mesenchymal transition (EMT) of RPE cells. **A** Schematic view of the EMT experiment. RPE cells were stimulated with human TGF-β2 with/without EVs. **B** The morphological changes of ARPE-19 and phRPE cells after TGF-β2 with/without MSC-EVs treatment in each group. Scale bar = 100 μm. **C** Quantitative analysis of RPE cell circularity/perimeter at day 0 and day 3. **D** The expression level of EMT marker genes (*ACTA2*, *FN1 *and *VIM*) and epithelial cell markers tight junction *ZO-1*, in RPE cells after TGF-β2 stimulation with/without MSC-EVs treatment. *18S* and *RPL11* were used as housekeeping genes for normalization. **E** Representative confocal images of immunofluorescence staining in TGF-β2-induced phRPE cells treated with/without EVs. Scale bar = 100 μm. Data presented as mean of fold change (2^−ΔΔCt) ± SD. n = 3 independent biological replicates. One-way ANOVA with Tukey’s multiple comparison test, *p<0.05, **p<0.01, ***p<0.001, ****p<0.0001

To understand the mechanisms underlying MCS-EV-mediated suppression of subretinal fibrosis, we investigated their effects on TGF-β1/2-induced EMT in both ARPE-19 and phRPE cells. A dose-dependent pilot experiment was conducted to identify the optimal EV concentrations, and we have since chosen a ratio of 1 cell vs 2000 EV particles in this study (Supplementary Fig. 1). The uptake of EVs by APRE-19 cells was visualized by confocal microscopy and flow cytometry analysis (Supplementary Fig. 2). Upon TGF-β stimulation, ARPE-19 cells adopted an elongated, fibroblast-like shape consistent with EMT, while phRPE cells were also observed to have large visible hole-like structures. MSC-EV treatment notably mitigated these morphological changes in both APRE-19 and phRPE cells. Specifically, the cell circularity was reversed from a spindle, myofibroblast morphology to a more rounded epithelial shape, similar to that of controls (Fig. 3B, C). Quantitative RT-PCR analysis revealed that MSC-EVs markedly suppressed TGF-β2-induced upregulation of EMT marker genes, including *ACTA2*, *FN1*, and *VIM*. The treatment also upregulated the epithelial tight junction marker gene, *ZO-1*, in TGF-β2-treated phRPE cells (Fig. 3D). The same response was observed in ARPE-19 cells (Supplementary Fig 3). The results were confirmed at the protein level by immunocytochemistry, evidenced by improved ZO-1 expression and reduced expression of vimentin, fibronectin, and α-SMA by MSC-EVs in TGF-β-treated phRPE cells (Fig. 3E). Our results suggest that MSC-EVs suppressed EMT in RPE cells.

### MSC-EVs suppressed the transition of macrophages into myofibroblasts in PMs

**Fig. 4:**
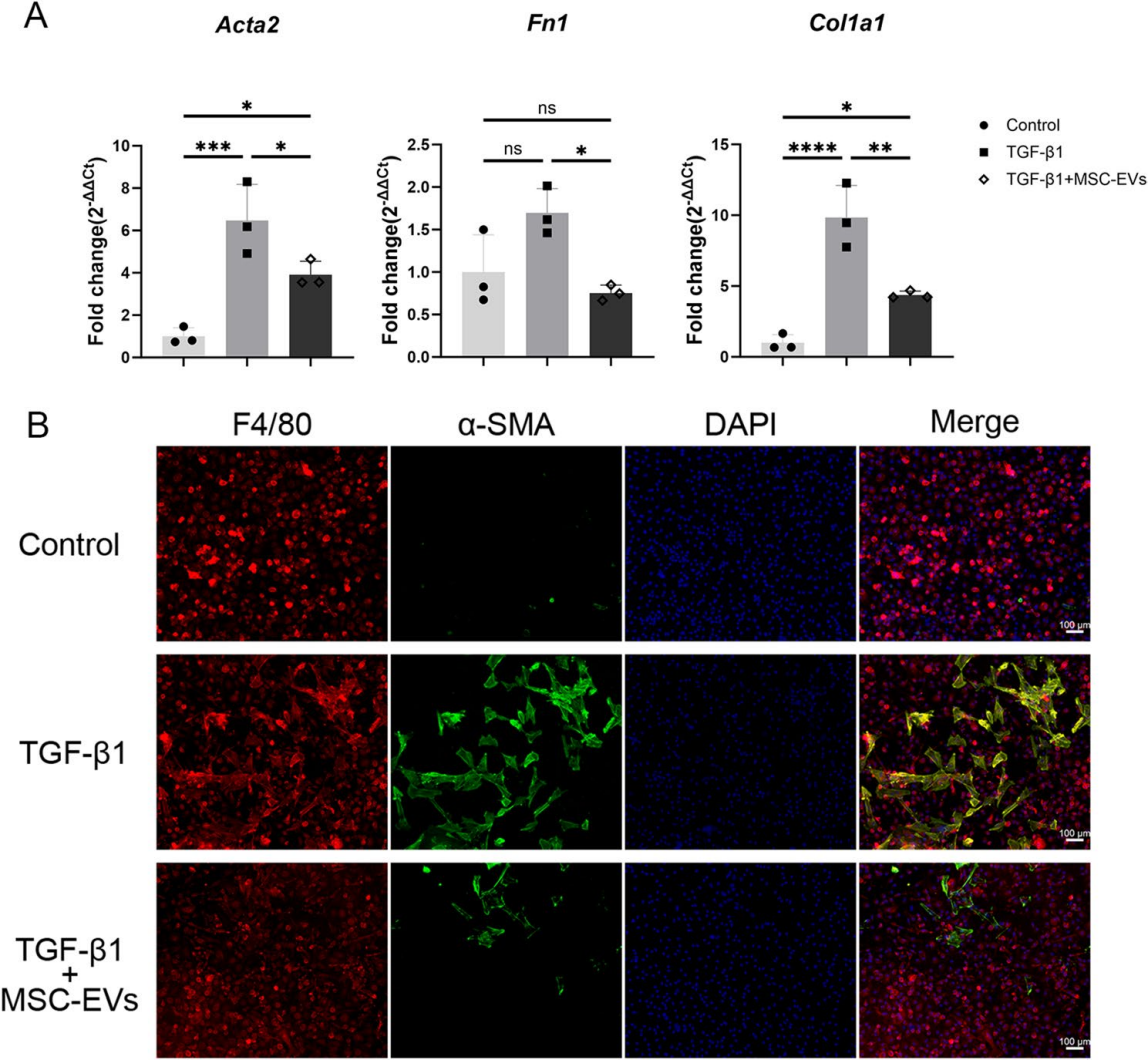
MSC-EVs inhibited transition of peritoneal macrophages (PMs) into myofibroblasts (MMT). Primary PMs were treated with mouse TGF-β1 and EVs simultaneously for 96 hours to detect the effect of EVs on TGF-β1-inducd MMT. **A** Expression level of myofibroblast cell marker genes (*Acta2*, *Fn1* and *Col1a1*) in different groups. Gapdh was used as housekeeping gene for normalization. **B** Representative confocal images of F4/80 and α-SMA immunofluorescence staining in TGF-β1-induced MMT with/without EVs treatment. Data presented as mean of fold change (2^−ΔΔCt) ± SD. n = 3 independent biological replicates. One-way ANOVA with Tukey’s multiple comparison test, *p<0.05, **p<0.01, ***p<0.001, ****p<0.0001

We previously showed that MMT contributed to subretinal fibrosis formation [36]. Herein, we assessed the effects of MSC-EVs on TGF-β1-induced MMT in peritoneal macrophages. TGF-β1 treatment markedly elevated the expression of *Acta2*, *Col1a1*, and *Fn1* in macrophages. The addition of MSC-EVs, at a ratio of cell: EV = 1:2000, significantly reduced TGF-β1-induced upregulation of *Acta2*, *Col1a1*, and *Fn1* (Fig. 4A). Immunocytochemistry further confirmed the reduction of α-SMA by MSC-EVs in TGF-β1-treated macrophages (Fig. 4B). Our results suggest that MSC-EVs inhibited MMT.

### MSC-EVs restrained the activation of microglia and macrophages

**Fig. 5:**
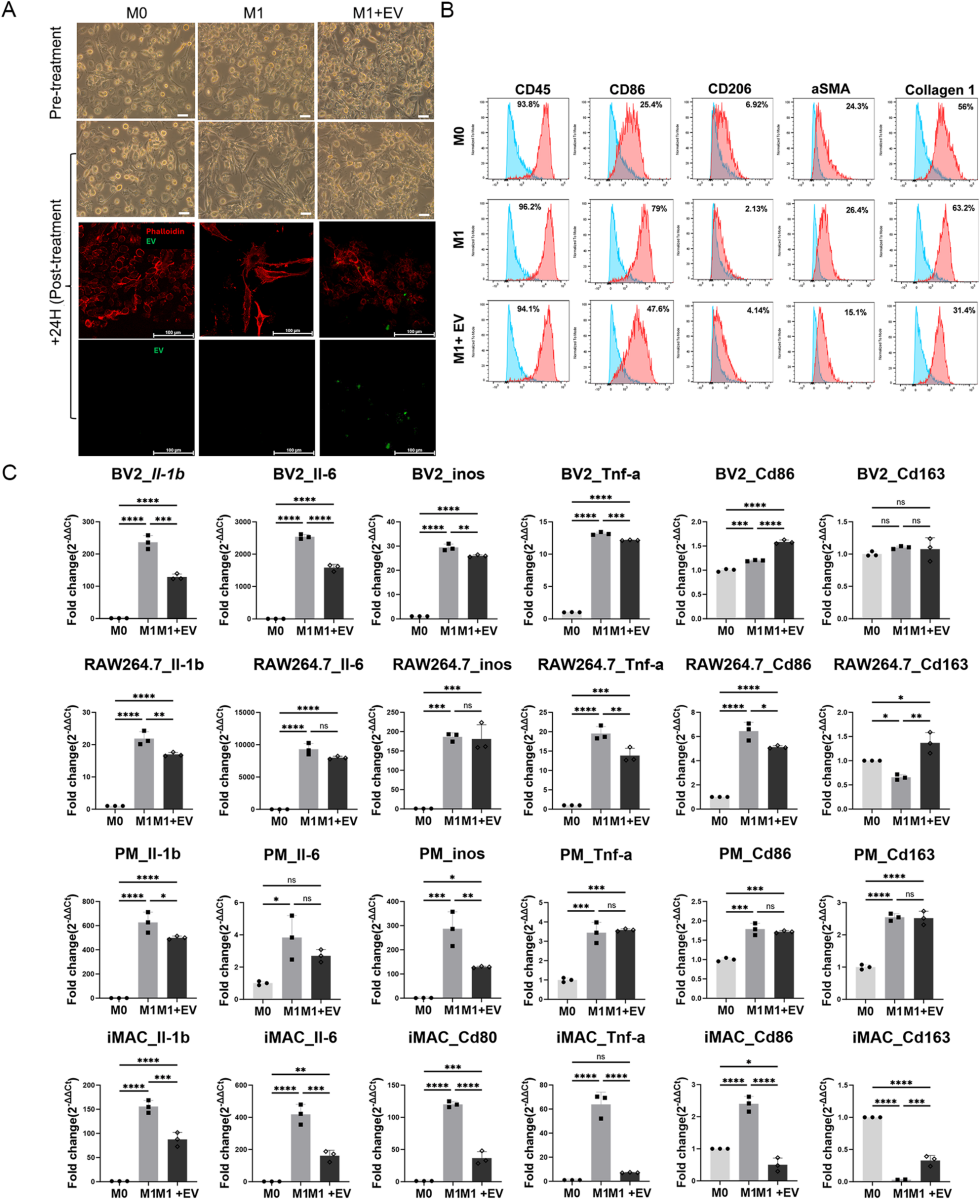
Effect of MSC-EVs on M1 polarization of microglia and macrophage cells. Cells were treated with LPS and IFN-γ to induce M1 polarization, and administrated MSC-EVs simultaneously. **A** The morphology of iMAC cells and MSC-EVs uptaking. Scale bar = 100 μm. **B** Flow cytometry of M1-associated markers in iMAC cells with/without MSC-EVs treatment. **C** Expression of M1 marker genes and pro-inflammation cytokines in different groups of BV2 cells, RAW264.7 cells, peritoneal macrophages (PMs), and iMAC cells. Gapdh was used as housekeeping gene for normalization. Data presented as mean of fold change (2^−ΔΔCt) ± SD. n = 3 independent biological replicates. One-way ANOVA with Tukey’s multiple comparison test, *p<0.05, **p<0.01, ***p<0.001, ****p<0.0001

Subretinal inflammation, particularly infiltrating macrophages, plays a crucial role in subretinal fibrosis [37], and MSC-EVs are known to possess immunomodulatory properties [38]. Next, we examined the effects of MSC-EVs on the activation of macrophages and microglia using the iMAC, RAW246.7 cells, peritoneal macrophages, and BV2 cells. Upon M1 stimulation, iMAC displayed an elongated morphology, which was reduced by MSC-EV treatment as shown by phase contrast and phalloidin immunostaining images (Fig. 5A). The expression of M1-associated marker, CD86 was significantly decreased following MSC-EV treatment (Fig. 5B). At the mRNA level, M1 stimulation upregulated the expression of *Il1b*,* Il6*,* Inos*,* Tnfa*, and *Cd86* in iMAC, RAW264.7, peritoneal macrophages, and BV2 microglia (Fig. 5C). MSC-EV treatment significantly reduced M1-induced upregulation of *Il6* and *Il1b* in all cell types, *Inos* in iMACs, peritoneal macrophages, and BV2 cells, and *Tnfa* and *Cd86* in iMACs, RAW264.7, and BV2 cells (Fig. 5C). Overall, our findings suggest that MSC-EVs attenuated LPS/IFN-γ-induced M1 polarization in macrophages and microglia.

### MSC-EVs reduced the accumulation of Iba-1^**+**^ cells in the lesion of subretinal fibrosis

**Fig. 6:**
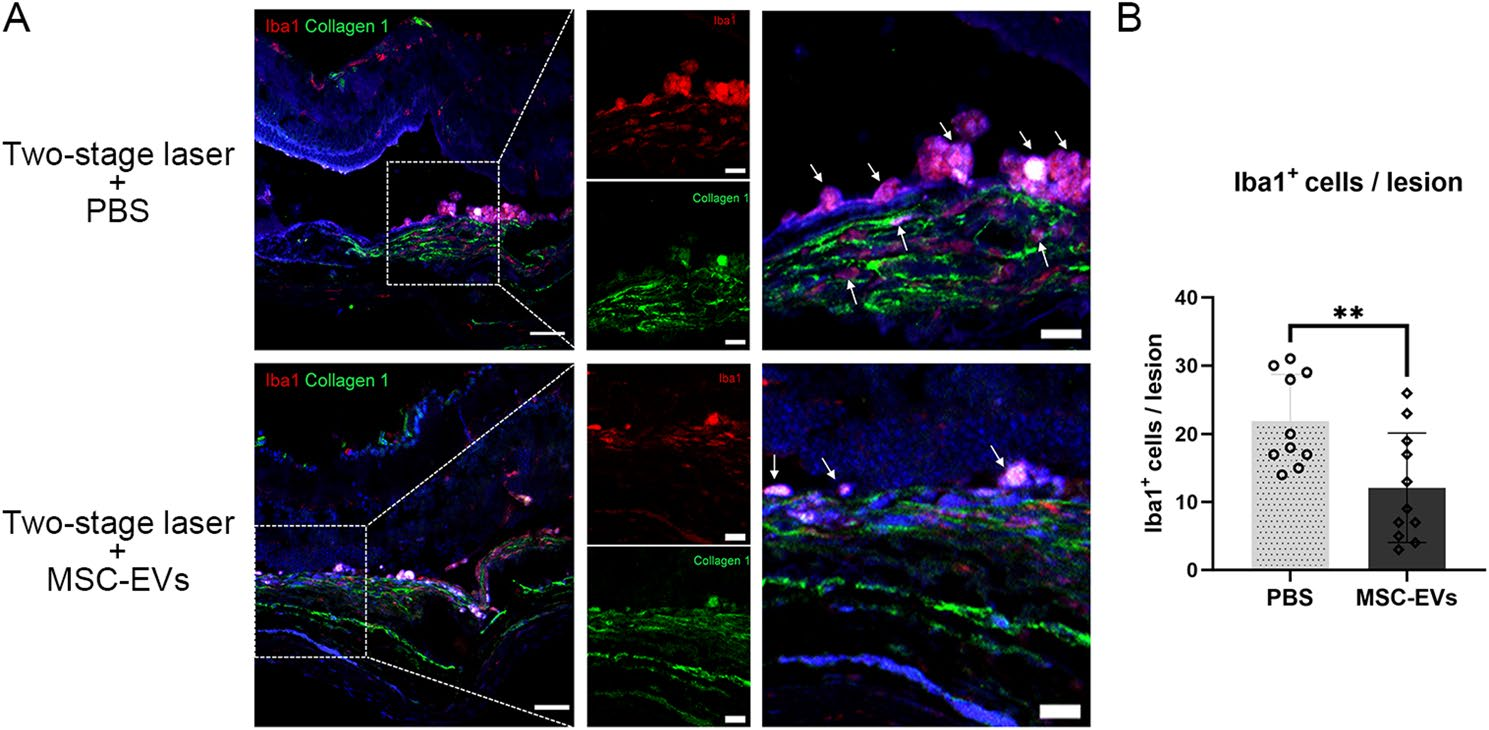
MSC-EVs suppressed the infiltration of Iba1^+^ cell in subretinal fibrotic lesion. **A** Representative immunofluorescence staining on frozen sections of mice retina with subretinal fibrosis from PBS control group and MSC-EV group stained for nucleus (blue), Iba1 (red) and collagen-1 (green). Sclar bar = 50 μm. Right images show magnified area of white box. Sclar bar = 20 μm. **B** Quantitative analysis of number of Iba1^+^ cells expressed per lesion in each group. Data shown as mean ± SD, n = 9 - 11 lesions per group. Independent Student’s t-test, **p<0.01

We further investigated the impact of MSC-EVs on immune cell infiltration in our two-stage laser-induced mouse model. Abundant accumulation of Iba1⁺ cells was observed within and surrounding the collagen-1⁺ fibrotic regions in PBS-treated mice (Fig. 6A). Intravitreal MSC-EV treatment reduced the number of Iba-1^+^ cells by 46% (from 21.9 ± 6.8 cells/lesion in PBS group to 12.1 ± 8.0 cells/lesion in MSC-EV group) (Fig. 6B).

### Cell-type-specific modulation of TGF-β1 downstream signaling by MSC-EVs

**Fig. 7:**
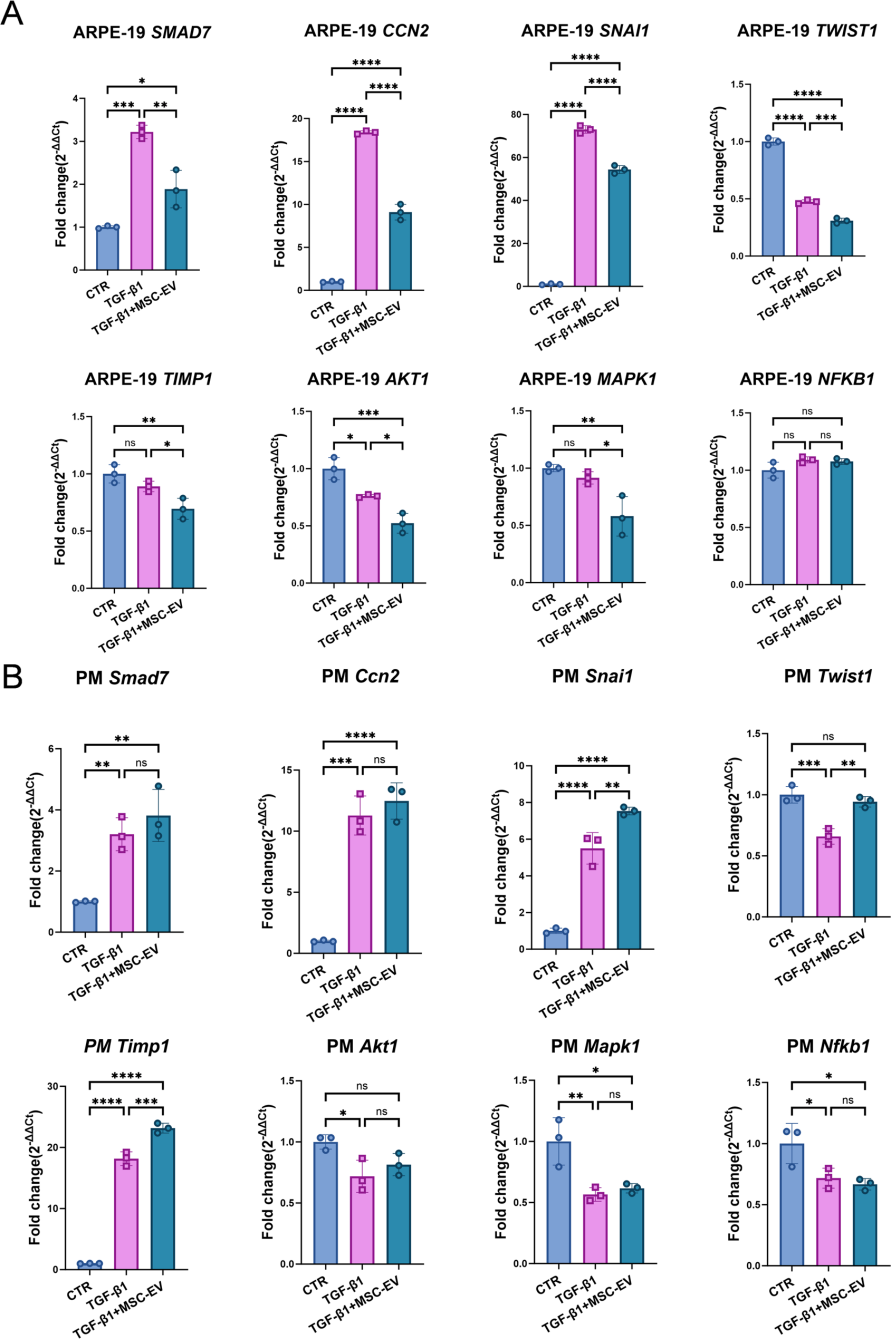
Differential and cell-type-specific modulation of TGF-β1 signaling by MSC-EVs in ARPE-19 EMT and PM MMT models. **A** - **B** The expression level of canonical and non-canonical TGF-β mediators (*SMAD7*, *CCN2*, *SNAI1*, *TIMP1*, *TWIST1*, *AKT1*, *MAPK1*, and *NFKB1*) in ARPE-19 and PM treated with or without MSC-EVs. Cells were stimulated with TGF-β1 alone or co-incubated with MSC-EVs. Data are presented as the mean ± SD of three independent biological replicates. One-way ANOVA with Tukey’s multiple comparison test. *p<0.05, **p<0.01, ***p<0.001, ****p<0.0001

To further delineate the downstream signaling cascades involved in MSC-EV-mediated suppression of TGF-β1-induced EMT and MMT, we quantified the transcriptional dynamics of canonical and non-canonical TGF-β signaling molecules. In the ARPE-19 EMT model, MSC-EVs potently abrogated TGF-β1-induced upregulation of *SMAD7*,* CCN2*, and *SNAI1*. Conversely, the expression of *TWIST1* and *AKT1* was diminished following TGF-β1 stimulation, and MSC-EV treatment further reduced their transcript levels compared to the TGF-β1-only group (Fig. 7A).

In the PM MMT model, while TGF-β1 upregulated *Smad7*,* Ccn2*,* Snai1*, and *Timp1*, and down-regulated *Twist1*,* Akt1*,* Mapk*, and *Nfkb1* expression, the introduction of MSC-EVs failed to reverse these broad transcriptional alterations. The sole exception was *Twist1*, which demonstrated a responsive modulation and was significantly reversed following MSC-EV treatment (Fig. 7B). Collectively, these data indicate that MSC-EVs comprehensively inhibit EMT in RPE cells by broadly rewiring the TGF-β signaling network. In contrast, their anti-fibrotic effects on macrophage phenotypic transition appear to be mediated through highly selective downstream nodes. This reveals a sophisticated, context-dependent regulatory mechanism, highlighting the profound cell-type specificity of MSC-EV function.

### Abundance distribution and functional prediction of MSC-EV miRNAs

**Fig. 8:**
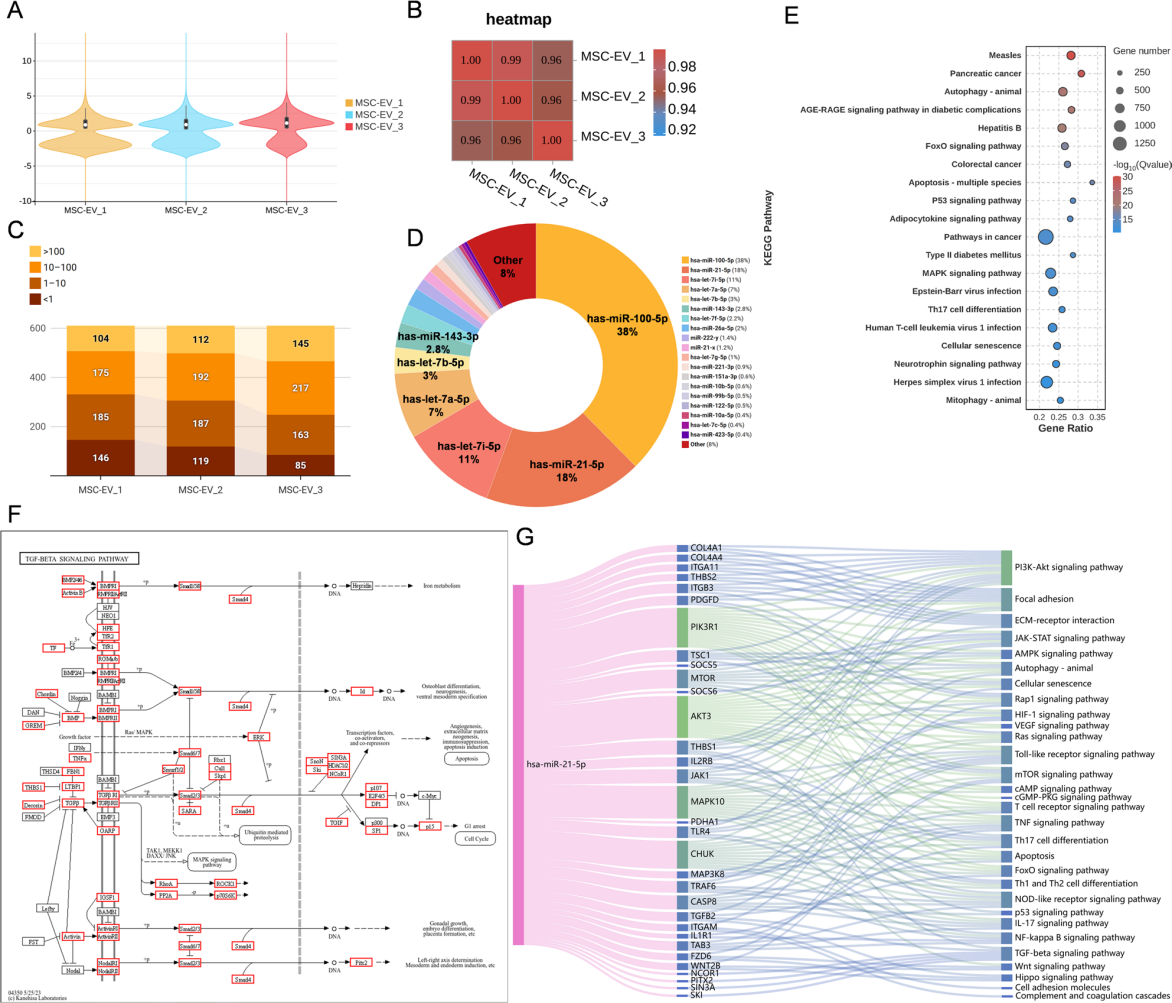
Abundance profiling and functional enrichment analysis of miRNAs sequenced from MSC-EVs. **A** Violin plot illustrating the consistency of the global miRNA expression distribution across the three MSC-EV samples. **B** Sample-to-sample correlation heatmap shows that the Pearson correlation coefficient > 0.95 indicates a high degree of overall correlation and strong biological reproducibility among the MSC-EV replicates. **C** Stacked columns plot displaying the distribution of miRNAs across different abundance ranges (<1, 1–10, 10–100, and >100 TPM) within MSC-EVs.** D** Pie chart depicting the relative abundance of the top-expressed miRNAs. Percentages represent the proportion of each specific miRNA’s mean TPM relative to the total miRNA expression pool in the MSC-EVs. **E** The top 20 significantly enriched KEGG signaling pathway for the target genes of all miRNAs. **F** The canonical KEGG pathway map for TGF-β signaling (ko04350). The specific target genes predicted to be modulated by the globally identified MSC-EV miRNA are explicitly highlighted with red boundary box. **G** A Sankey diagram delineating the regulatory interactions of a single dominant candidate, hsa-miR-21-5p. The visualization displays the multi-dimensional flow of information from the miRNA (left node) to its diverse target mRNAs (middle nodes), and subsequently to their enriched functional assignment to downstream KEGG pathways (right nodes)

To identify the specific molecular effectors responsible for the anti-fibrotic effects of MSC-EVs, we profiled their miRNA cargo using small RNA sequencing. The data demonstrated highly reproducibility and quality, with a consistent expression distribution and a strong correlation (*R* > 0.95) across all biological replicates (Fig. 8A, B). A total of 610 miRNAs were identified. While the majority of miRNAs exhibited a TPM range between 1 and 100, a small fraction (17%-23%) were highly expressed (TPM > 100, Fig. 8C). Notably, two of these highly enriched, miR-100-5p and miR-21-5p account for 56% of the total miRNA pool (Fig. 8D). KEGG pathway enrichment analysis of their predicted target genes was significantly enriched for pathways related to the MAPK signaling and Th17 cell differentiation, suggesting these miRNAs may contribute to the anti-fibrotic effects by modulating inflammatory and fibrotic signaling cascades (Fig. 8E).

KEGG pathway analysis from our dataset also identified the TGF-β signaling pathway (ko04350) as a major significantly pathway enriched among the predicted targets of MSC-EV-derived miRNAs. This is visually mapped in Fig. 8F, signaling nodes with a substantial density in this cascade (highlighted by red boundary boxes) are designated as targets of the identified miRNAs, referring to the canonical SMAD2/3 axis, noncanonical MAPK/ERK axis and TGFβ receptor inhibition. Further, the multi-dimensional network analysis reveals that miR-21-5p converges on the TGF-β signaling pathway by concurrently targeting a multi-gene network comprising *NCOR1*,* PITX2*,* SIN3A*,* SKI*,* TGFB2*, and *THBS1*. In addition, miR-21-5p modulates PI3K-Akt, JAK-STAT axis via targeting *AKT3*,* JAK1*,* PIK3R1*,* IL2RB*, and *SOCS5* (Fig. 8G). ECM-receptor interaction and focal adhesion were also regulated by miR-21-5p through common *COL4A1*,* COL4A4*,* ITGA11*,* ITGB3*,* THBS1*, and *THBS2*. These findings suggest that MSC-EVs exploit the pleiotropic nature of miR-21-5p to regulate the TGF-β cascade. This is accomplished by simultaneous intervention at multiple nodes, including transcriptional effectors and signal-transducers.

### MSC-EVs miR-21-5p dynamically suppressed TGF-β1-induced mesenchymal transitions and attenuated inflammation

**Fig. 9:**
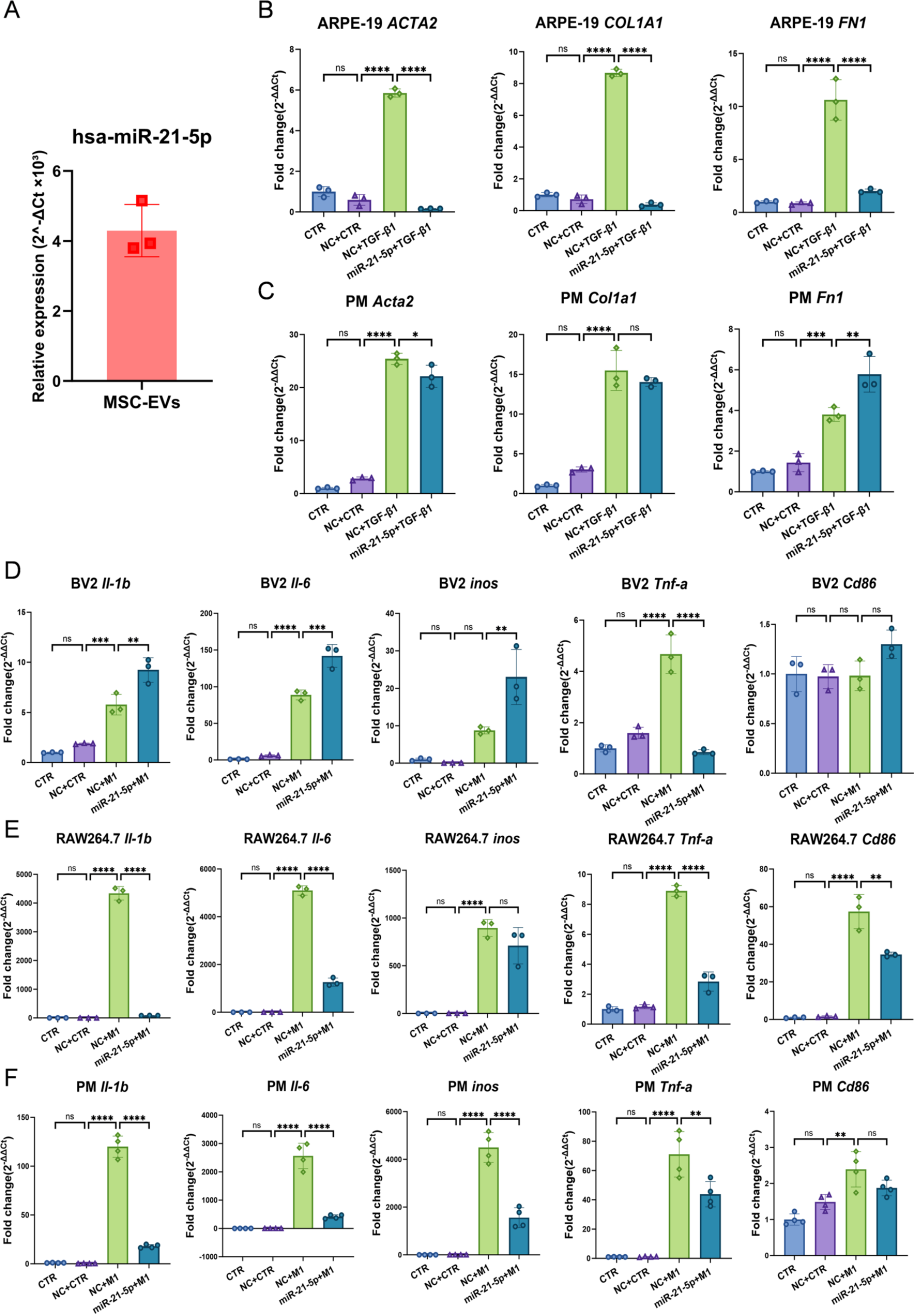
Validation of MSC-EV miR-21-5p abundance and its modulatory effects on TGF-β-induced mesenchymal transitions and M1 polarization induced by LPS and IFN-γ. **A** RT-qPCR analysis verifying the medium-to-high expression level of miR-21-5p in MSC-EVs. Relative expression was calculated using the 2^-ΔCt method and normalized to the exogenous spike-in cel-miR-39. Values were scaled by 10³ for optimal visualization. **B** - **C** The relative expression level of *ACTA2*, *COL1A1*, and *FN1* in ARPE-19 cells and PMs. Cells were transfected with either a miR-21-5p mimic or a mimic Negative Control (NC) and subsequently stimulated with TGF-β1 to induce EMT and MMT, respectively. **D** - **F** The expression of M1 polarization-associated pro-inflammatory genes (*Il-1b*, *Il-6*, *inos*, *Tnf-a*, and *Cd86*) in BV2, RAW264.7 and PMs. Cells were transfected with either a miR-21-5p mimic or a mimic NC and subsequently stimulated with LPS and IFN-γ to induce M1 polarization. Data are presented as the mean ± SD. n = 3independent biological replicates. One-way ANOVA with Tukey’s multiple comparison test. *p<0.05, **p<0.01, ***p<0.001, ****p<0.0001

To functionally validate our small RNA sequencing data, we first confirmed that hsa-miR-21-5p was highly expressed and abundantly packaged within the MSC-EVs (Fig. 9A). We then assessed its therapeutic potential using miR-21-5p mimic in two distinct fibrosis models. In ARPE19 cells, the miR-21-5p mimic abrogated TGF-β1-driven upregulation of pro-fibrotic markers, including *ACTA2*,* COL1A1*, and *FN1* (Fig. 9B). Furthermore, miR-21-5p mimic significantly inhibited the TGF-β1-induced expression of *CCN2* in ARPE-19 cells (Supplementary Fig. 4). In contrast, its effects in macrophages were more selective, while miR-21-5p overexpression attenuated the TGF-β1-induced upregulation of *Acta2*, it did not significantly affect the expression of *Col1a1* and *Fn1* (Fig. 9C). These findings functionally validate our bioinformatic predictions and revealed that MSC-EV-derived miR-21-5p exerts potent, yet highly selective, anti-fibrotic effects—comprehensively reversing EMT in epithelial cells while selectively modulating specific myofibroblast markers during macrophage phenotypic transition.

The immunomodulatory potential of miR-21-5p was confirmed by its consistent anti-inflammatory effects across multiple cell types. In BV2 microglia, RAW264.7, and PMs, overexpression of miR-21-5p mimic significantly attenuated LPS+IFNγ-induced TNF expression (Fig. 9D-F). In RAW264.7 cells, the mimic also profoundly suppressed the expression of *Il-1b*,* Il-6*, and *Cd86*, while in PMs, it mitigated the expression of *Il-1b*,* Il-6*, and *inos*. Collectively, these findings offer compelling in vitro evidence that MSC-EV-derived miR-21-5p is a potent and conserved negative regulator of M1 polarization in both macrophages and microglia.

## Discussion

In this study, we found that MSC-EVs significantly reduced subretinal fibrosis in a two-stage laser-induced mouse model. The therapeutic effect was more pronounced when the particles were delivered intravitreally compared to retroorbital injection. Mechanistically, MSC-EVs inhibited EMT and MMT in a cell-specific manner, suppressed the inflammatory response in microglia and macrophages, and reduced immune cell infiltration through miR-21-5p. The results highlight the promising role of MSC-EVs as a novel cell-free therapeutic strategy for subretinal fibrosis.

MSC-EVs have emerged as a potent therapeutic strategy for diverse fibrotic and inflammatory conditions. Their efficacy has been largely documented in non-neuronal tissues such as the liver, lung, heart, and skin, where they attenuate pathological ECM deposition, suppress inflammation, and promote tissue repair [22–25]. These pleiotropic effects are attributed to the delivery of bioactive cargos, including microRNAs, proteins, and lipids, which can modulate key cellular pathways [21], such as the TGF-β signaling axis, and reduce myofibroblast differentiation and activation [39, 40]. They can also modulate tissue immune response, for example, by polarizing macrophages from the proinflammatory M1 phenotype toward the anti-inflammatory M2 phenotype, thereby mitigating fibrogenesis [41–43]. This multi-targeted action may contribute to the therapeutic effect of MSC-EV in organ fibrosis.

Macular subretinal fibrosis in nAMD is the conversion of the choroidal neovascular membrane into a fibro-vascular lesion, a pathological remodeling fundamentally driven by chronic inflammation [44–48]. The two-stage laser-induced murine model of subretinal fibrosis mirrors the clinical progression closely. The second laser causes leakage and subretinal inflammation in pre-existing CNV, which mirrors recurrent macular oedema or hemorrhage in nAMD patients. Within this highly inflammatory microenvironment, the inflammatory mediators and infiltrating immune cells are the principal catalysts for fibrogenesis. Both microglia and macrophages accumulated at the subretinal fibrosis lesion, although only macrophages, not microglia, critically contribute to the development of fibrosis [37]. The diseased macula supplies ample molecular cues to recruit and activate choroidal fibroblasts [49], circulating fibrocytes [33], and to induce EMT and MMT. For example, complement fragment C5a can induce EMT of RPE cells [11], whereas C3a can drive MMT in infiltrating macrophages [36]. Moreover, macrophages can also exacerbate fibrosis by releasing multiple profibrotic mediators, including macrophage elastase (MMP12) [12], VEGF, Ang-2, and uPAR [33].

We found that MSC-EVs act as a dual-blockade against fibrosis, concurrently suppressing both RPE-derived EMT and macrophage-derived MMT through distinct, cell-type-specific mechanisms. In the EMT model, MSC-EVs abrogated TGF-β-induced upregulation of *SNAI1* and *CCN2* - key drivers of phenotypic switching and matrix overproduction, respectively, thereby dismantling the complete fibrotic effector network in epithelial cells [50–54]. Conversely, in the MMT model, MSC-EVs failed to reverse the broad transcriptional reprogramming induced by TGF-β in macrophages, suggesting a more targeted, potentially non-canonical mechanism of action. This context-dependent versatility highlights the therapeutic promise of MSC-EVs: they simultaneously extinguish the inflammatory response in microglia and macrophages while dismantling the specific transcriptional hubs necessary for myofibroblast transformation.

MicroRNA profiling of MSC-EV identified miR-21-5p as a highly enriched transcript with predicted targets in immunomodulation and fibrosis. Although endogenous miR-21 is often described as a effector targeting SMAD7 [55] and a pro-fibrotic driver in idiopathic pulmonary fibrosis [56] and kidney fibrosis [57, 58], MSC-EV-derived miR-21 has been reported to exert opposing anti-fibrotic effects [59–61]. Our functional assays align with this protective role, demonstrating that MSC-EV-derived miR-21-5p suppresses TGF-β-induced EMT and MMT and attenuates pro-inflammatory cytokine production in macrophages and microglia.

This apparent paradox reflects the context-dependent and cell-specific regulatory nature of miR-21-5p. In an inflammatory microenvironment, exosomal miR-21-5p exerts anti-inflammatory effects by steering macrophages and microglia away from the M1 phenotype, in part via STAT3 signaling axis [62, 63]. Within the immune-infiltrated microenvironment of subretinal fibrosis, MSC-EV may therefore leverage this immunomodulatory function. By simultaneously suppressing upstream inflammation and rewiring the downstream TGF-β signaling, MSC-EV-derived miR-21-5p effectively disrupts the pathological feedback loop driving fibrogenesis.

The optimal delivery route remains a critical translational hurdle for MSC-EV therapy. Intravenous injection, though minimally invasive, results in rapid EV clearance by mononuclear phagocytes [64] and sequestration in the liver, lung, and spleen [65, 66], limiting its utility to systemic application or first-pass organ targeting (i.e., liver, lung, and spleen). For retinal diseases, the blood-retinal barrier (BBR) further precludes efficient systemic delivery. Considering that subretinal fibrosis in nAMD originates from CNV, i.e., the choroidal tissue, we compared two local delivery strategies: retroorbital (twice) and intravitreal injections. Despite using twice the MSC-EV dose, the retroorbital group achieved only ~ 65% of the efficacy of a single intravitreal injection (30% vs. 46% reduction in fibrotic lesion size). This suggests that only a limited amount of MSC-EVs injected into the orbital sinus reached the subretinal area, likely due to uptake by extraocular cells or diversion into orbital/choroidal circulations. These data demonstrate that retroorbital administration, although less invasive, is suboptimal for retinal MSC-EV delivery.

The robust therapeutic efficacy of MSC-EVs in our two-stage laser-induced subretinal fibrosis model supports their potential as a cell-free intervention, particularly for anti-VEGF refractory patients. Unlike live cell therapies, MSC-EVs possess inherent safety advantages, including low immunogenicity, absence of risks such as immune rejection and tumorigenicity. Nonetheless, clinical translation requires overcoming key bioengineering challenges, such as scalable manufacturing (scalability) and batch-to-batch heterogeneity. To ensure reproducibility and accelerate clinical adoption, future studies must adhere to the Minimal Information for Studies of Extracellular Vesicles 2023 (MISEV2023) guidelines [67]. Standardized, MISEV2023-compliant protocols for EV isolation, characterization, and quality control will be essential for establishing reproducible dosing regimens and realizing the therapeutic potential of MSC-EVs for retinal fibrosis.

The study has several limitations. While we functionally validated miR-21-5p, additional miRNAs identified in our sequencing may exert synergistic therapeutic effects, warranting further multifactorial investigations. The complete signaling network through which miR-21-5p regulates in EMT, MMT and inflammation also remains to be fully elucidated. Second, the short-term in vivo follow-up limits our ability to assess long-term efficacy and safety. Critical parameters, including off-target effects, biodistribution, and long-term ocular responses, have yet to be evaluated. Addressing these gaps will be crucial for advancing MSC-EV therapy toward clinical application.

## Conclusions

This study provides compelling evidence that MSC-EVs, via miR-21-5p cargo, attenuate subretinal fibrosis through a multi-pronged mechanism: suppressing inflammatory responses, inhibiting RPE-derived EMT, and blocking macrophage-originated MMT. This integrated neuro-immune and anti-fibrotic blockade positions MSC-EVs as a versatile cell-free therapeutic platform with significant potential for treating subretinal fibrosis.

## Supplementary Information

Supplementary Material 1: Supplementary Figure 1: Determination of optimal MSC-EV dosage based on EV particles concentration gradient experiment. ARPE-19 cells were stimulated with TGF-β2 following with/without EVs treatment at different cell:EV particles ratio. A - C Quantitative analysis of ARPE-19 cell circularity from day 0 to day 3 after EV exposure with increasing concentrations. The minimum effective MSC-EV dose identified in this assay (green box and blue font) was selected for subsequent vitro experiments. Data are presented as the mean ± SD. n = 3 independent biological replicates.

Supplementary Material 2: Supplementary Figure 2: MSC-EVs internalization and dose-dependent uptake by RPE. A MSC-EVs were uptaken by RPE. EVs were fluorescently labeled in green (Supplementary Table 2), and tagged EVs were administered to RPE within 1-2 H. Internalization of tagged EVs by RPE cells was observed through RPE staining with Rhodamine Phalloidin visualizing actin filaments (red) (Supplementary Table 3). Z-stack images were acquired using confocal microscopy and processed with Imaris imaging analysis software version 10.2 to generate a 3D rendering, illustrating EV internalization within the cells. B Cross section view of EV internalization. RPE cells stained with Rhodamine Phalloidin can be observed showing MSC-EVs (shown in green) within the cell boundaries. C Representative images of EV (green) uptaken by ARPE-19 cells. D 3-D histogram showed the fluorescence in ARPE-19 treated with TGF-β2 and different EV particle ratio.

Supplementary Material 3: Supplementary Figure 3: MSC-EVs decreased the genes expression in ARPE-19 stimulated with TGF-β1.The expression level of EMT marker genes (*ACTA2*, *FN1*, *COL1A1*, and *VIM*), epithelial cell markers tight junction gene (*TJP1/ZO-1*), and *TGFB1*, *VEGFA* in ARPE-19 cells after TGF-β1 stimulation with/without MSC-EVs treatment. GAPDH was used as a housekeeping gene for normalization. Data presented as mean of fold change (2^−ΔΔCt) ± SD. n = 3 independent biological replicates. One-way ANOVA with Tukey’s multiple comparison test, *p<0.05, **p<0.01, ***p<0.001, ****p<0.0001.

Supplementary Material 4: Supplementary Figure 4: The modulation effect of miR-21-5p on TGF-β signaling in EMT and MMT. A - B The expression level of canonical and non-canonical TGF-β mediators in ARPE-19 and PM cells treated with or without miR-21-5p. Cells were transfected with either a miR-21-5p mimic or a mimic Negative Control (NC) and subsequently stimulated with TGF-β1 to induce EMT and MMT, respectively. Data are presented as the mean ± SD. n = 3 independent biological replicates. One-way ANOVA with Tukey’s multiple comparison test, *p<0.05, **p<0.01, ***p<0.001, ****p<0.0001.

Supplementary Material 5.

Supplementary Material 6.

Supplementary Material 7.

## Data Availability

All data supporting the findings of this study are available within the article and its Supplementary Materials. Additional datasets generated and/or analyzed during the current study are available from the corresponding author upon reasonable request.

## Abbreviations

AMD: Age-related macular degeneration
nAMD: Neovascular age-related macular degeneration
MSC-EVs: Mesenchymal stem cell-derived extracellular vesicles
RPE: Retinal pigment epithelial
EMT: Epithelial-mesenchymal transition
MMT: Macrophage-to-myofibroblast transition
ECM: Extracellular matrix
iMACs: IPCS-derived macrophages
phRPE: Primary human RPE cells
PMs: Peritoneal macrophages
