# Supplementary figures and images for: MSC-EVs attenuate subretinal fibrosis in choroidal neovascularization through miR-21-5p-mediated inhibition of EMT and MMT and suppression of inflammation
Source: J Neuroinflammation. 2026 Apr 30;23:218. doi: 10.1186/s12974-026-03836-w (PMC13312753; doi:10.1186/s12974-026-03836-w)

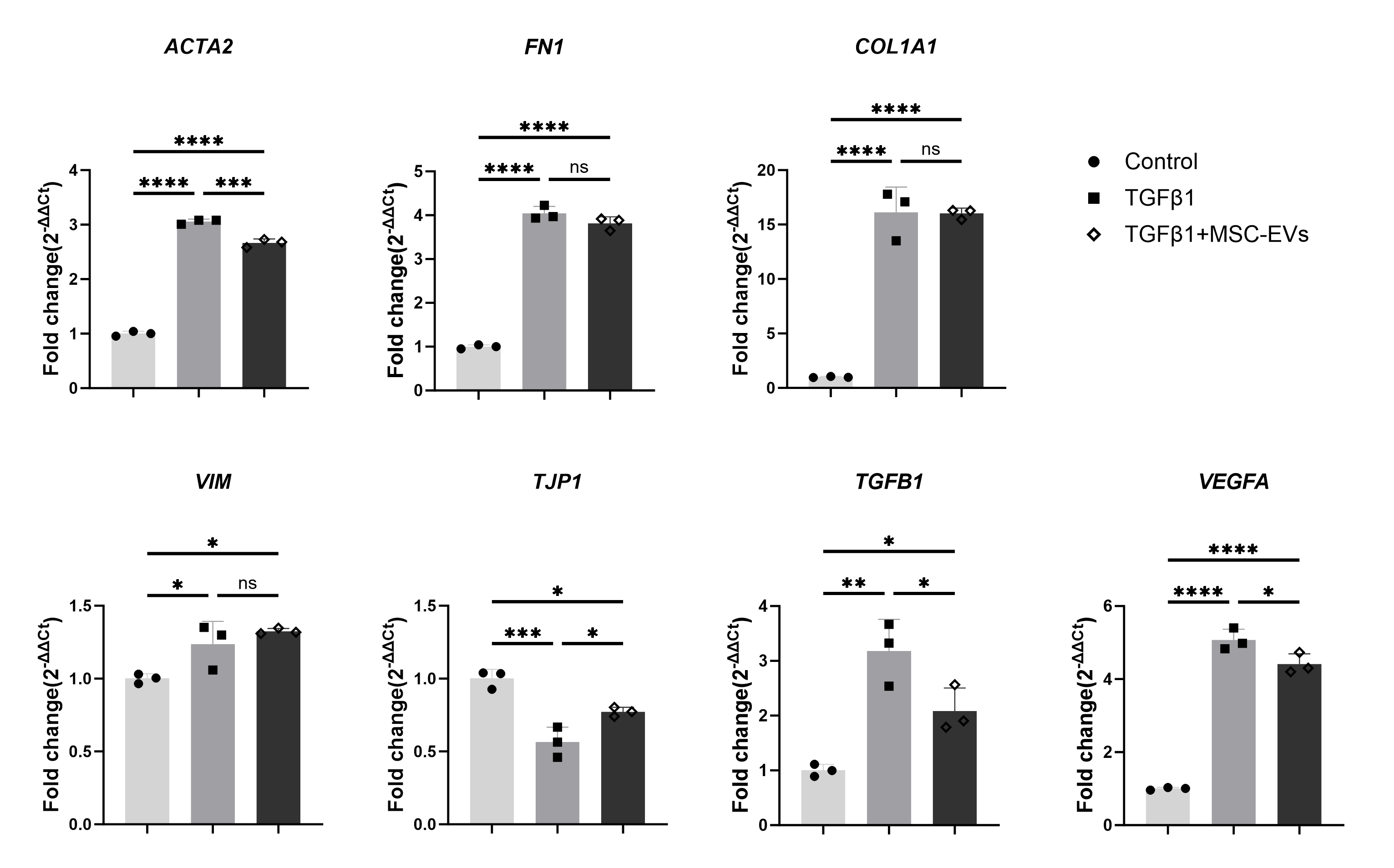

Supplement: Supplementary file 1 — Supplementary Material 1: Supplementary Figure 1: Determination of optimal MSC-EV dosage based on EV particles concentration gradient experiment. ARPE-19 cells were stimulated with TGF-β2 following with/without EVs treatment at different cell:EV particles ratio. A - C Quantitative analysis of ARPE-19 cell circularity from day 0 to day 3 after EV exposure with increasing concentrations. The minimum effective MSC-EV dose identified in this assay (green box and blue font) was selected for subsequent vitro experiments. Data are presented as the mean ± SD. n = 3 independent biological replicates. [file 12974_2026_3836_MOESM1_ESM.tif]

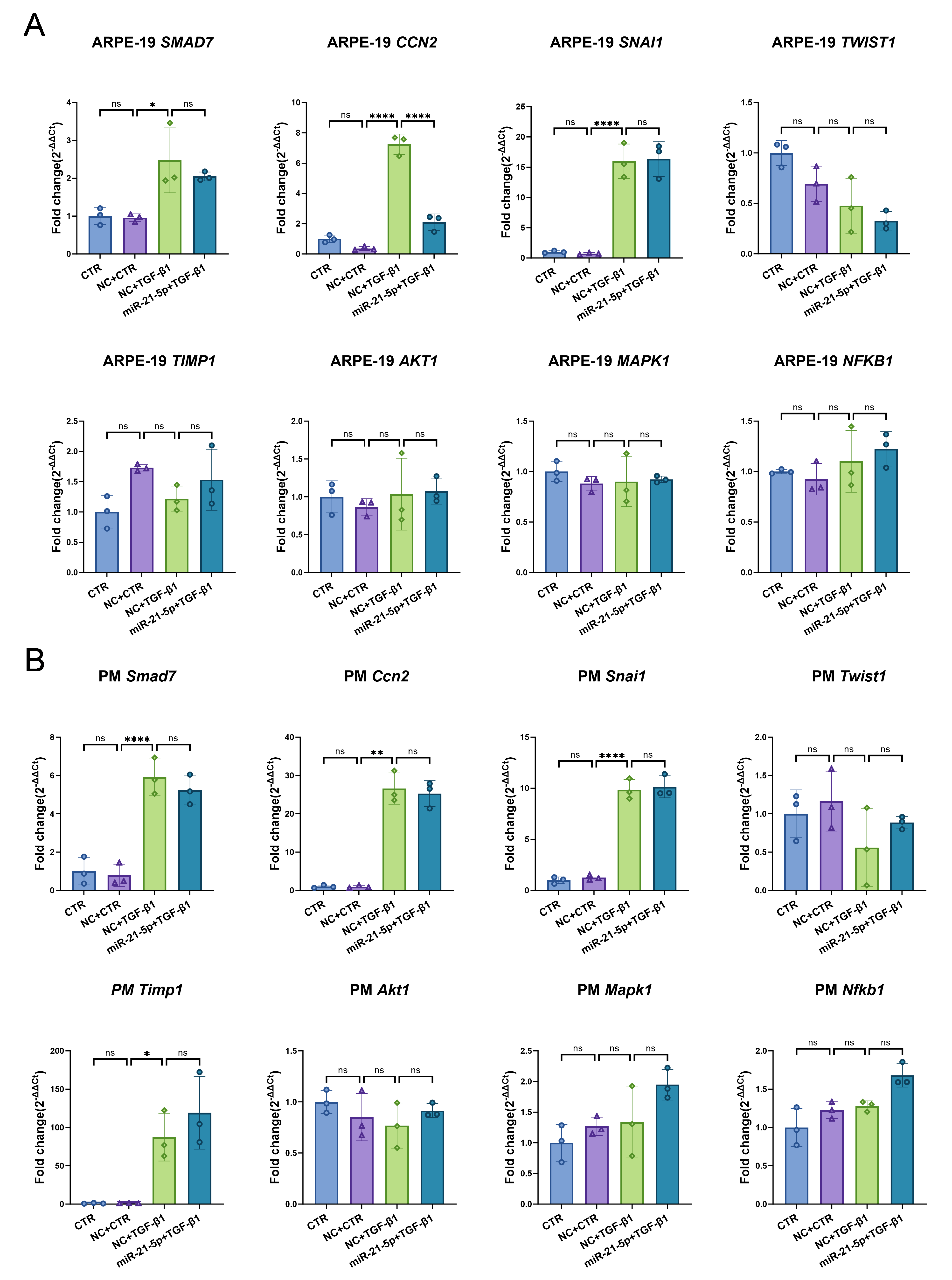

Supplement: Supplementary file 2 — Supplementary Material 2: Supplementary Figure 2: MSC-EVs internalization and dose-dependent uptake by RPE. A MSC-EVs were uptaken by RPE. EVs were fluorescently labeled in green (Supplementary Table 2), and tagged EVs were administered to RPE within 1-2 H. Internalization of tagged EVs by RPE cells was observed through RPE staining with Rhodamine Phalloidin visualizing actin filaments (red) (Supplementary Table 3). Z-stack images were acquired using confocal microscopy and processed with Imaris imaging analysis software version 10.2 to generate a 3D rendering, illustrating EV internalization within the cells. B Cross section view of EV internalization. RPE cells stained with Rhodamine Phalloidin can be observed showing MSC-EVs (shown in green) within the cell boundaries. C Representative images of EV (green) uptaken by ARPE-19 cells. D 3-D histogram showed the fluorescence in ARPE-19 treated with TGF-β2 and different EV particle ratio. [file 12974_2026_3836_MOESM2_ESM.tif]

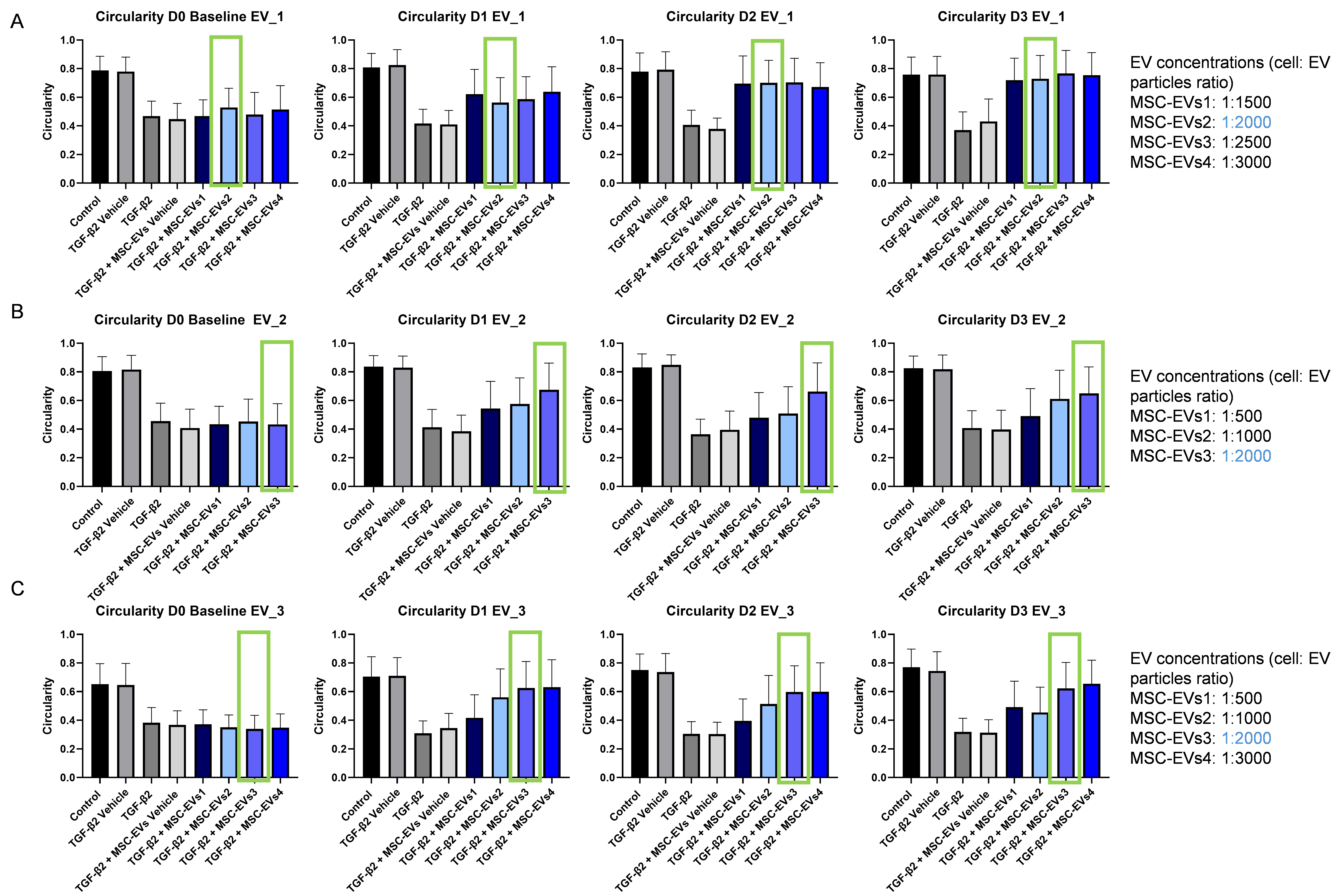

Supplement: Supplementary file 3 — Supplementary Material 3: Supplementary Figure 3: MSC-EVs decreased the genes expression in ARPE-19 stimulated with TGF-β1.The expression level of EMT marker genes (ACTA2, FN1, COL1A1, and VIM), epithelial cell markers tight junction gene (TJP1/ZO-1), and TGFB1, VEGFA in ARPE-19 cells after TGF-β1 stimulation with/without MSC-EVs treatment. GAPDH was used as a housekeeping gene for normalization. Data presented as mean of fold change (2^−ΔΔCt) ± SD. n = 3 independent biological replicates. One-way ANOVA with Tukey’s multiple comparison test, *p<0.05, **p<0.01, ***p<0.001, ****p<0.0001. [file 12974_2026_3836_MOESM3_ESM.tif]

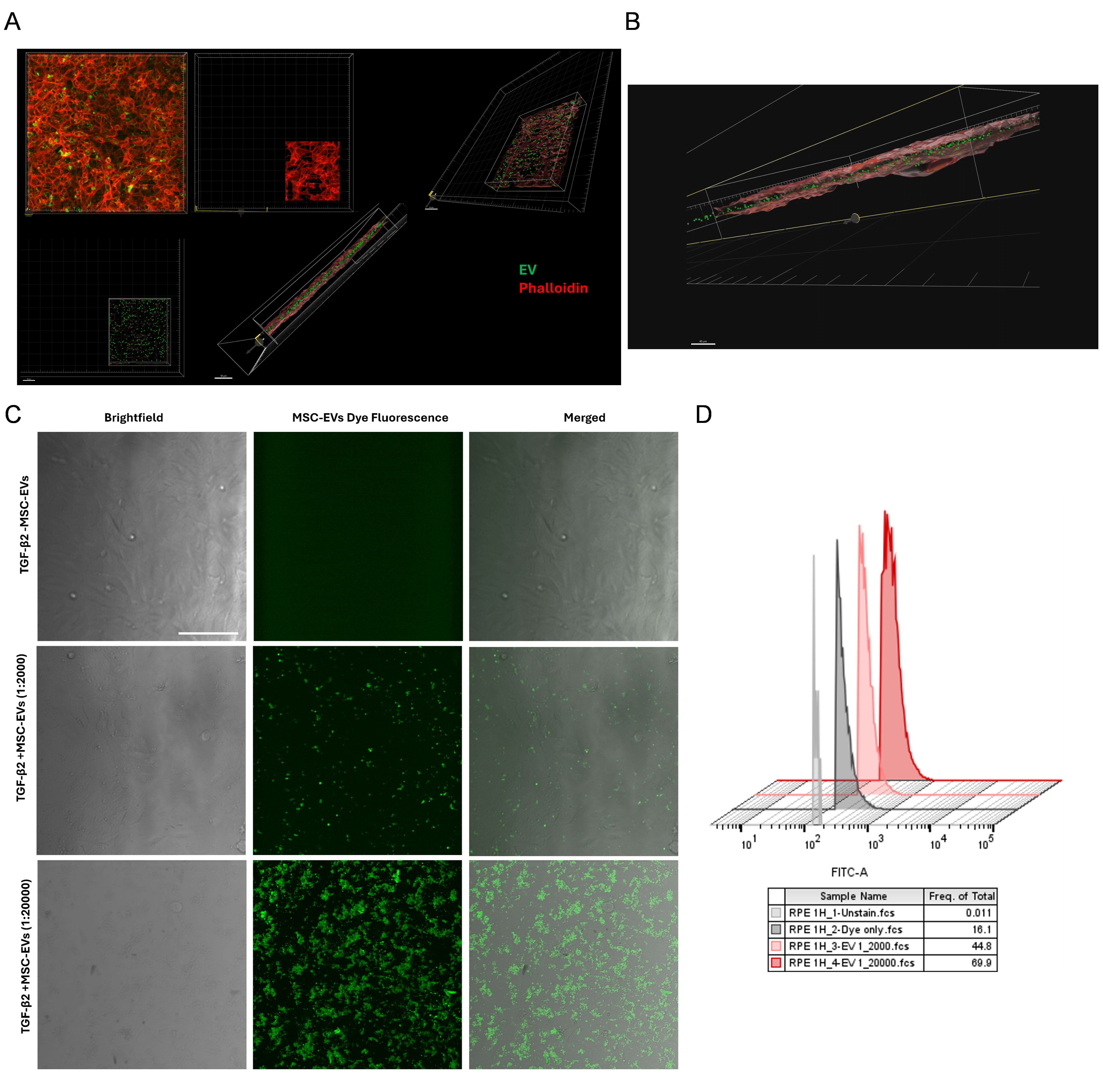

Supplement: Supplementary file 4 — Supplementary Material 4: Supplementary Figure 4: The modulation effect of miR-21-5p on TGF-β signaling in EMT and MMT. A - B The expression level of canonical and non-canonical TGF-β mediators in ARPE-19 and PM cells treated with or without miR-21-5p. Cells were transfected with either a miR-21-5p mimic or a mimic Negative Control (NC) and subsequently stimulated with TGF-β1 to induce EMT and MMT, respectively. Data are presented as the mean ± SD. n = 3 independent biological replicates. One-way ANOVA with Tukey’s multiple comparison test, *p<0.05, **p<0.01, ***p<0.001, ****p<0.0001. [file 12974_2026_3836_MOESM4_ESM.tif]
