# Supplementary material for: MSC-EVs attenuate subretinal fibrosis in choroidal neovascularization through miR-21-5p-mediated inhibition of EMT and MMT and suppression of inflammation
Source: J Neuroinflammation. 2026 Apr 30;23:218. doi: 10.1186/s12974-026-03836-w (PMC13312753; doi:10.1186/s12974-026-03836-w)
Supplement: Supplementary file 5 — Supplementary Material 5. [file 12974_2026_3836_MOESM5_ESM.docx]

| **Supplementary Table 1.** Primer sequences of genes for quantitative RT-PCR |
| --- |

| Genes |  | Forward |  | Reverse |
| --- | --- | --- | --- | --- |
| *Il-1β* |  | TGGACCTTCCAGGATGAGGACA | | GTTCATCTCGGAGCCTGTAGTG |
| *Il-6* |  | TCTGCAAGAGACTTCCATCCAGT | | TCTGCAAGTGCATCATCGTTGT |
| *inos* |  | GGCAAACCCAAGGTCTACGTT | | TCGCTCAAGTTCAGCTTGGT |
| *Tnf-α* |  | GGTGCCTATGTCTCAGCCTCTT | | GCCATAGAACTGATGAGAGGGAG |
| *Cd86* |  | ACGTATTGGAAGGAGATTACAGCT | | TCTGTCAGCGTTACTATCCCGC |
| *Cd163* | | AGACACACGGAGCCATCAAAA | | TGGACAAACCTTTTACAACCAGGA |
| *Acta2* |  | TGGCACCACTCTTTCTATAACG | | GGTCATTTTCTCCCGGTTGG |
| *Col1a1* | | GTGGCGGTTATGACTTCAGC | | GGCTGCGGATGTTCTCAATC |
| *Fn1* |  | GATTGGGAGGAAGAAGACAGATGA | | TTGAACTGTGGAGGGAACATCC |
| *Gapdh* | | ACTTTGTCAAGCTCATTTCC | | TGCAGCGAACTTTATTGATG |
| *Smad7* | | ACGCAGGAGGAAAGACCAG | | ATCCACGAGTTACATCCCCC |
| *Ccn2* |  | CACAGTGCCAGAATGCACAC | | TCTCGCTAGAGCAGGTCTGT |
| *Snai1* |  | ATGGAGTGCCTTTGTACCCG | | TGAGGGAGGTAGGGAAGTGG |
| *Twist1* | | CCGTGGACAGAGATTCCCAG | | TGGCTGATTGGCAAGACCTC |
| *Timp1* |  | TCGTGGGCTCTGAGGACTAC | | AGTTTTTCCTGGGGGAAGGC |
| *Mapk1* | | CCCAAGTGATGAGCCCATTG | | CTTACACCATCTCTCCCTTGCT |
| *Akt1* |  | CCGCCTGATCAAGTTCTCCTA | | TGCCCACAGTAGAAACATCCTC |
| *Nfkb1* |  | CCTGCTTCTGGAGGGTGATG | | CAGCCTTCTCCCAAGAGTCG |
| *ACTA2* | | AAAAGACAGCTACGTGGGTGA | | GCCATGTTCTATCGGGTACTTC |
| *COL1A1* | | TTGGATGGTGCCAAGGGAG | | CAGTAGCACCATCATTTCCACG |
| *FN1* |  | CGGTGGCTGTCAGTCAAAG | | AAACCTCGGCTTCCTCCATAA |
| *TJP1* |  | CAACATACAGTGACGCTTCACA | | CACTATTGACGTTTCCCCACTC |
| *TGFB1* | | TACCTGAACCCGTGTTGCTCTC | | GTTGCTGAGGTATCGCCAGGAA |
| *VEGFA* | | TTGCCTTGCTGCTCTACCTCCA | | GATGGCAGTAGCTGCGCTGATA |
| *SMAD7* | | AAGAGGCTGTGTTGCTGTGA | | ACAGCATCTGGACAGTCAGT |
| *CCN2* |  | CATCTTCGGTGGTACGGTGT | | AACCACGGTTTGGTCCTTGG |
| *SNAI1* | | TCGGAAGCCTAACTACAGCGA | | AGATGAGCATTGGCAGCGAG |
| *TWIST1* | | GTCCGCAGTCTTACGAGGAG | | GCTTGAGGGTCTGAATCTTGCT |
| *TIMP1* | | CTGTTGTTGCTGTGGCTGAT | | ACGCTGGTATAAGGTGGTCTG |
| *MAPK1* | | ACACCAACCTCTCGTACATCGG | | TGGCAGTAGGTCTGGTGCTCAA |
| *AKT1* |  | AGCGACGTGGCTATTGTGAAG | | GCCATCATTCTTGAGGAGGA |
| *NFKB1* | | GCAGCACTACTTCTTGACCACC | | TCTGCTCCTGAGCATTGACGTC |
| *18S* | | GTAACCCGTTGAACCCCATT | | CCATCCAATCGGTAGTAGCG |
| *RPL11* | | AGAGTGGAGACAGACTGACGCG | | CGGATGCCAAAGGATCTGACAG |
| *GAPDH* | | GGAAGGTGAAGGTCGGAGTC | | GATCTCGCTCCTGGAAGATGG |
| *cel-miR-39* | | CGGGTGTAAATCAGCTTGAA | | |
