# Supplementary material for: MSC-EVs attenuate subretinal fibrosis in choroidal neovascularization through miR-21-5p-mediated inhibition of EMT and MMT and suppression of inflammation
Source: J Neuroinflammation. 2026 Apr 30;23:218. doi: 10.1186/s12974-026-03836-w (PMC13312753; doi:10.1186/s12974-026-03836-w)
Supplement: Supplementary file 6 — Supplementary Material 6. [file 12974_2026_3836_MOESM6_ESM.docx]

| **Supplementary Table 2.** Primary antibodies used in the study | | | |  |
| --- | --- | --- | --- | --- |
| Primary antibodies | Cat. No. | Company | Host | Dilution |
| CoraLite® Plus 488-conjugated ZO-1 Polyclonal antibody | CL488-21773 | Proteintech | Rabbit | 1:200 |
| CoraLite®555-conjugated Vimentin Recombinant antibody | CL555-80232 | Proteintech | Rabbit | 1:200 |
| CoraLite® Plus 647-conjugated smooth muscle actin specific Monoclonal antibody | CL647-67735 | Proteintech | Rabbit | 1:200 |
| Anti-Collagen Type I Antibody | AB758 | Sigma-Aldrich | Goat | 1:50 |
| Griffonia Simplicifolia Lectin I (GSL I) Isolectin B4, Biotinylated | B-1205-.5 | Vector Laboratories | | 1:50 |
| CD31 Monoclonal Antibody | MA1-40074 | Invitrogen | Rat | 1:200 |
| Anti-Collagen I + Collagen III antibody | ab34710 | Abcam | Rabbit | 1:200 |
| Anti-Iba1 antibody | ab283346 | Abcam | Rat | 1:400 |
| Anti-F4/80 antibody | ab6640 | Abcam | Rat | 1:200 |
| Anti-alpha smooth muscle Actin antibody | ab7817 | Abcam | Mouse | 1:200 |
| DAPI | C0065 | Solarbio |  | 1:1000 |
| Anti-Hu Fc Receptor Binding Inhibitor | 14-9161-73 | Invitrogen |  | 1:200 |
| Tra-1-60 | A24882 | Thermo Fisher Scientific | Mouse | 1:50 |
| CD9 Rabbit mAb | 13174 | Cell Signaling Technology | Rabbit | 1:1000 |
| CD63 Polyclonal antibody | 25682-1-AP | Proteintech | Rabbit | 1:1000 |
| TSG101 Polyclonal Antibody | 28283-1-AP | Proteintech | Rabbit | 1:2000 |
| ExoGlow-Protein EV Labeling Kit | EXOGP300A-1 | Cambridge Bioscience |  | 1:500 |
| Flow antibodies |  |  |  |  |
| FITC anti-human CD45 | 304006 | BioLegend |  | 5 µL per 1 x 10^6^ cells |
| PE Mouse Anti-Human CD206 | 555954 | BD Pharmingen |  | 5 µL per 1 x 10^6^ cells |
| Anti-Collagen Type I-FITC Antibody | FCMAB412F | Merck |  | 5 µL per 1 x 10^6^ cells |
| Anti-Hu CD86 Alexa Fluor 488 | 53-0869-42 | Invitrogen |  | 5 µL per 1 x 10^6^ cells |
