# Supplementary material for: MSC-EVs attenuate subretinal fibrosis in choroidal neovascularization through miR-21-5p-mediated inhibition of EMT and MMT and suppression of inflammation
Source: J Neuroinflammation. 2026 Apr 30;23:218. doi: 10.1186/s12974-026-03836-w (PMC13312753; doi:10.1186/s12974-026-03836-w)
Supplement: Supplementary file 7 — Supplementary Material 7. [file 12974_2026_3836_MOESM7_ESM.docx]

| **Supplementary Table 3.** Second antibodies used in the study | | |  |
| --- | --- | --- | --- |
| Second antibodies | Cat. No. | Company | Dilution |
| Alexa Fluor 594-conjugated goat anti-rat IgG | A11007 | Invitrogen | 1:400 |
| Alexa Fluor 488-conjugated goat anti-rabbit IgG | A11034 | Invitrogen | 1:400 |
| Alexa Fluor 488 goat anti-mouse IgG | A11029 | Invitrogen | 1:400 |
| Alexa Fluor® 488 AffiniPure™ Donkey Anti-Goat IgG (H+L) | 705-545-147 | Jackson ImmunoResearch | 1:400 |
| Alexa Fluor® 594 Streptavidin | 016-580-084 | Jackson ImmunoResearch | 1:200 |
|  |  |  |  |
| Rhodamine phalloidin | R415 | Invitrogen | 1:500 |
| Anti-rabbit IgG, HRP-linked Antibody | 7074 | Cell signaling technology | 1:2000 |
